# TRPC1 links calcium signaling to cellular senescence in the protection against posttraumatic osteoarthritis

**DOI:** 10.1172/jci.insight.182103

**Published:** 2024-12-24

**Authors:** Meike Sambale, Starlee Lively, Osvaldo Espin-Garcia, Pratibha Potla, Chiara Pastrello, Sarah Bödecker, Linda Wessendorf, Simon Kleimann, Peter Paruzel, Rojiar Asgarian, Alexandra Tosun, Johanna Intemann, Jessica Bertrand, Francesco Dell’Accio, Mohit Kapoor, Thomas Pap, Joanna Sherwood

**Affiliations:** 1 Institute of Musculoskeletal Medicine, University Hospital Münster, Münster, Germany.; 2 Osteoarthritis Research Program, Division of Orthopaedics, Schroeder Arthritis Institute, and; 3 Krembil Research Institute, University Health Network, Toronto, Ontario, Canada.; 4 Department of Epidemiology and Biostatistics, University of Western Ontario, London, Ontario, Canada.; 5 Dalla Lana School of Public Health and Department of Statistical Sciences, University of Toronto, Toronto, Ontario, Canada.; 6 Department of Biostatistics, University Health Network, Toronto, Ontario, Canada.; 7 Department of Orthopaedic Surgery, Otto-von-Guericke University Magdeburg, Magdeburg, Germany.; 8 Experimental Medicine and Rheumatology, Queen Mary University of London, London, United Kingdom.

**Keywords:** Bone biology, Cell biology, Calcium channels, Cellular senescence, Osteoarthritis

## Abstract

Transient receptor potential channel 1 (TRPC1) is a widely expressed mechanosensitive ion channel located within the endoplasmic reticulum membrane, crucial for refilling depleted internal calcium stores during activation of calcium-dependent signaling pathways. Here, we have demonstrated that TRPC1 activity is protective within cartilage homeostasis in the prevention of cellular senescence–associated cartilage breakdown during mechanical and inflammatory challenge. We revealed that TRPC1 loss is associated with early stages of osteoarthritis (OA) and plays a nonredundant role in calcium signaling in chondrocytes. *Trpc1^–/–^* mice subjected to destabilization of the medial meniscus–induced OA developed a more severe OA phenotype than WT controls. During early OA development, *Trpc1^–/–^* mice displayed an increased chondrocyte survival rate; however, remaining cells displayed features of senescence including p16^INK4a^ expression and decreased Sox9. RNA-Seq identified differentially expressed genes related to cell number, apoptosis, and extracellular matrix organization. *Trpc1^–/–^* chondrocytes exhibited accelerated dedifferentiation, while demonstrating an increased susceptibility to cellular senescence. Targeting the mechanism of TRPC1 activation may be a promising therapeutic strategy in OA prevention.

## Abstract 2

Loss of the ion channel TRPC1 during early osteoarthritis leads to increased cellular senescence and accelerated joint damage.

## Graphical abstract

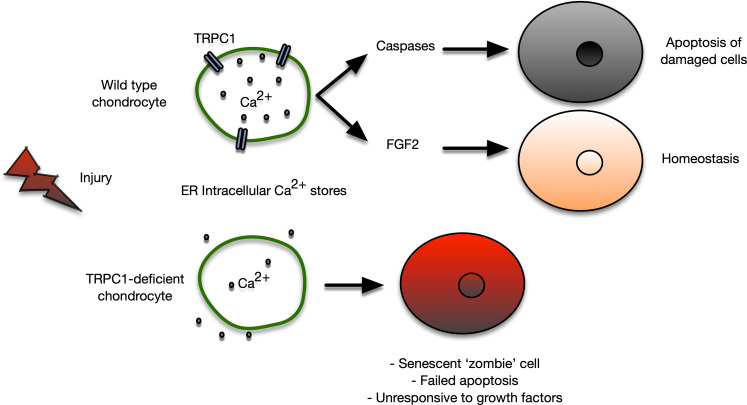

## Introduction

Osteoarthritis (OA) is a chronic degenerative joint disorder featuring severe destruction of the articular cartilage, subchondral bone remodeling, and localized inflammation that causes pain, disability, and reduced quality of life to millions of people worldwide (1, 2). No approved disease modifying OA drug (DMOAD) options are currently available; therefore, OA progression is first managed via the administration of NSAIDs and physiotherapy, before ultimately being managed by joint arthroplasty. Loss of chondrocyte phenotypic stability is a hallmark of OA development, whereby the chondrocytes situated within the cartilage extracellular matrix (ECM) ultimately fail in their compensatory expression of key anabolic markers, such as Sox9 and type II collagen, and increase their expression of hypertrophic and catabolic markers, such as inflammatory cytokines, type X collagen, and matrix metalloproteinases (MMPs) (3, 4).

The involvement of ion channels in OA-mediated processes has been identified, yet the role of such channels and their mechanisms of activity remain understudied in OA research (5). Disregulation of calcium signaling pathways resulting in alterations to local electrostatic fields and protein synthesis (6) has been implicated to lead to increased expression of catabolic enzymes including MMP13, alongside an increase in cell death (7, 8). Controlled oscillation of intracellular Ca^2+^ concentrations is a highly conserved phenomenon found in many different cell types. They are required for modulating downstream gene expression profiles and various cellular processes, including those involved in maintaining a stable chondrocyte phenotype through the regulation of expression of key genes including Sox9 and aggrecan (9, 10). Transient receptor potential canonical 1 (TRPC1) channel is a broadly expressed member of the TRP superfamily of cation channels that is normally situated within the endoplasmic reticulum (ER) membrane. TRPC1 channels can form heteromeric complexes with other TRP channels or with stromal interaction molecule (STIM) and Orai, key players in store-operated calcium entry (SOCE) (11, 12). Activation upon mechanical or receptor-driven depletion of intracellular Ca^2+^ stores leads to activation of STIM1, which in turn activates TRPC1 and Orai1 channels to allow influx of extracellular Ca^2+^ into the cell, driving the controlled Ca^2+^ oscillation cycles.

While cyclic shear and compressive forces are required for normal articular cartilage development and homeostasis (13), OA is exacerbated by the increased force exerted on cartilage following biomechanical alterations and early disease-associated matrix changes. Mechanical stimulation generated from cyclic forces, for example during typical weight-bearing movement, stimulates repeated Ca^2+^ influx activity within the chondrocytes (14). Activation of mechanoreceptors in chondrocytes including TRPV4 and PIEZO2 has been demonstrated to trigger anabolic and homeostatic responses, demonstrating the potential for ion channels to be targeted within the search for potential DMOAD therapies (15–17). While little is known about the specific role of TRPC1 in chondrocytes, deficiency of *Trpc1* in mice has been observed to limit skeletal muscle contraction during sustained activity, suggesting that TRPC1 may be required to sufficiently and repeatedly refill intracellular stores during prolonged stimulation (18).

Here we investigate the hypothesis that TRPC1 plays a functional role within cartilage homeostasis during the refilling of intracellular Ca^2+^ stores required to maintain controlled intracellular Ca^2+^ oscillations triggered by biomechanical and biochemical signaling in the joint. We examine how OA development influences the expression of TRPC1 and how the loss of TRPC1 leads to reduced chondrocyte phenotypic stability and increased cartilage damage within the destabilization of the medial meniscus (DMM) model of surgically induced OA. Our data suggest that the loss of TRPC1 during early OA predisposes the cartilage to an increased cellular senescence–linked phenotype triggered by both mechanical and inflammatory challenge.

## Results

### TRPC1 is expressed in human and murine articular cartilage, is reduced during OA development, and is functionally nonredundant.

TRPC channels are known to mediate an intracellular Ca^2+^ response to mechanical force; therefore, considering the role of mechanical stress as a predominant driver in OA development, we compared the expression of TRPC channels in patients with OA within a previously published microarray set (GSE114007) (19). At mRNA level, *TRPC1* was the most highly expressed TRPC channel in both normal and OA cartilage (Supplemental Figure 1A; supplemental material available online with this article; https://doi.org/10.1172/jci.insight.182103DS1).

**Figure 1:**
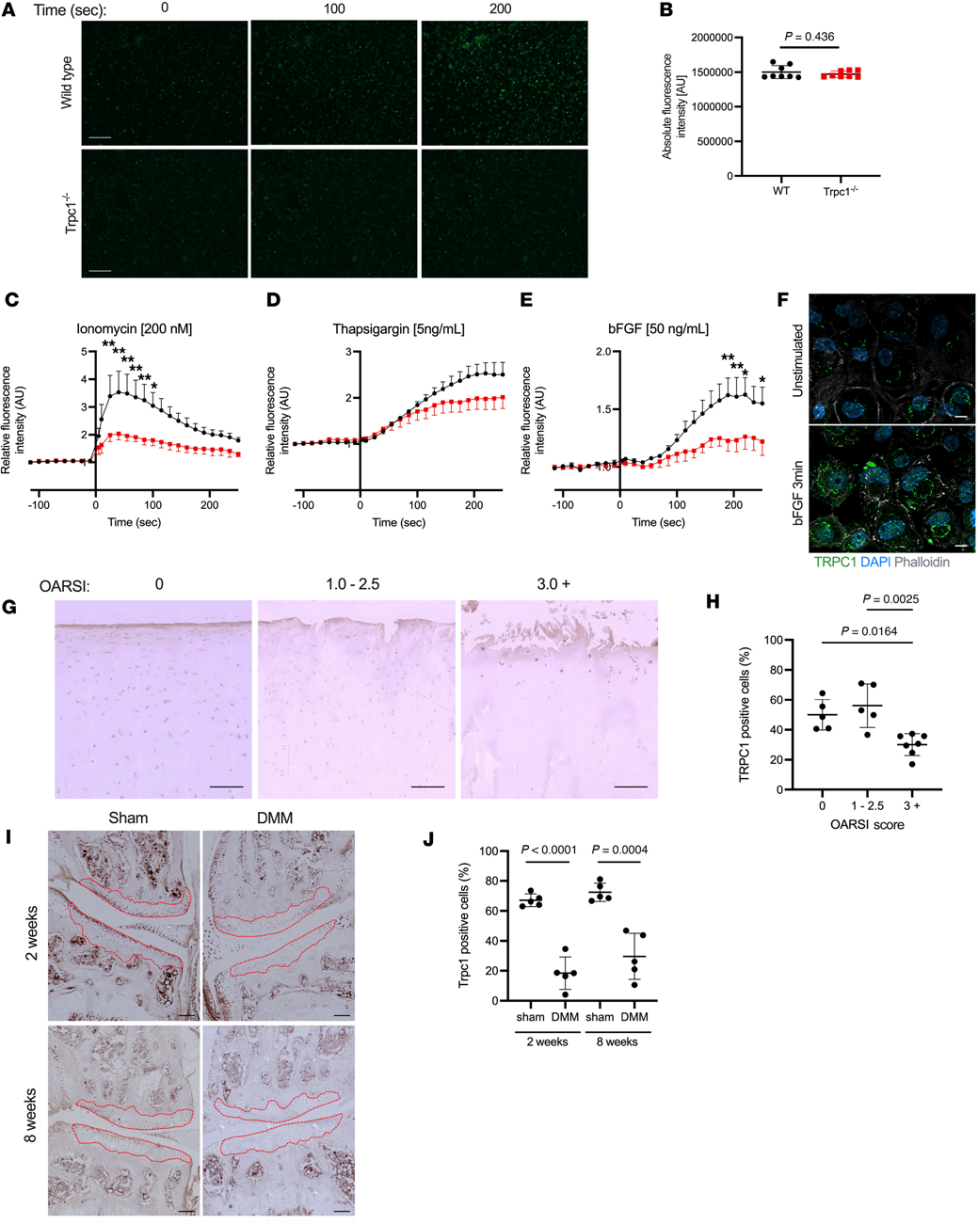
Analysis of TRPC1 protein expression in human and murine articular cartilage. (**A**) Fluorescence images depicting intracellular Ca^2+^ (green) within early passage WT and TRPC1^–/–^ chondrocytes loaded with Fluo-4 Ca^2+^ indicator, during stimulation with 5 ng/mL thapsigargin. Scale bar: 200 μm. (**B**) Comparison of basal intracellular Ca^2+^ levels as measured by fluorescence intensity of early passage WT and *Trpc1^–/–^* chondrocytes. Unpaired *t* test (*n* = 8). (**C**–**E**) Time course analysis of intracellular Ca^2+^ levels as measured by fluorescence intensity of loaded Fluo-4 Ca^2+^ indicator in WT and *Trpc1^–/–^* chondrocytes during stimulation with either ionomycin (200 nM), thapsigargin (5 ng/mL), or bFGF (50 ng/mL). Two-way ANOVA with multiple comparisons (*n* = 4). *P < 0.05 and **P < 0.01. (**F**) Immunofluorescence detection of TRPC1 (green) in WT murine chondrocytes in resting conditions and 3 minutes following bFGF (50ng/mL) stimulation. Cell cytoskeleton is counterstained with phalloidin (white) and nuclei with DAPI (blue). Scale bar: 20 μm. For isotype negative control staining, see Supplemental Figure 3A. (**G**) Immunohistological detection of TRPC1 in human cartilage sections comparing healthy (OARSI score 0), early OA (OARSI score 1.0–2.5) and advanced OA (OARSI score 3.0+). Scale bar: 100 μm. For isotype negative control staining, see Supplemental Figure 3B. (**H**) Graph showing quantification of TRPC^+^ cells within the cartilage expressed as percentage of total number of cells identified (*n* = 4 participants). One-way ANOVA with Tukey’s multiple-comparison test. (**I**) Immunohistological detection of TRPC1 in murine knee joint 2 weeks and 8 weeks following sham control or DMM surgery. Dotted red outline indicates area used for analysis of articular cartilage. Scale bar: 100 μm. For isotype negative control staining, see Supplemental Figure 3C. (**J**) Quantification of the relative number of TRPC1^+^ cells within the tibial articular cartilage expressed as a percentage of cells present *P* values: unpaired *t* test (*n* = 5 mice).

We hypothesized that TRPC1 is required in chondrocytes during the refilling of intracellular Ca^2+^ stores following and during activation of signaling pathways. In order to assess how the lack of TRPC1 affects the ability to respond to biochemical stimuli known to drive intracellular Ca^2+^ release, intracellular calcium measurements were collected before and following addition of stimuli, such as thapsigargin, to WT and *Trpc1^–/–^* chondrocytes using a Fluo-4 fluorescent intracellular indicator (Figure 1A). WT and *Trpc1^–/–^* chondrocytes were crucially found to have an equal level of intracellular calcium mobilization in resting conditions (Figure 1B). PBS, used as a control for the mechanical disturbance caused by the addition of liquid into the resting medium, did not produce a release of intracellular calcium in either genotype (Supplemental Figure 2). In contrast, ionomycin, an ionophore known to facilitate the transfer of Ca^2+^ into cells, led to an increase in intracellular calcium in both genotypes; however, this mobilization was tempered significantly in *Trpc1^–/–^* chondrocytes compared with in controls (Figure 1C). Thapsigargin, an inhibitor of ER ATPase Ca^2+^ pumps, causes Ca^2+^ to be released from intracellular stores (20). Stimulation of WT chondrocytes led to a relatively long-lived increase in intracellular calcium; however, this again appears stunted in *Trpc1^–/–^* chondrocytes (Figure 1D). bFGF, a physiological stimuli involved in mechanically induced biochemical signaling in chondrocytes (21), drove an increase in intracellular Ca^2+^ concentrations in WT cells, which was found to be significantly lower in *Trpc1^–/–^* chondrocytes (Figure 1E). This analysis demonstrates that the function of TRPC1 cannot be fully compensated for, suggesting that TRPC1 is an important and nonredundant channel in chondrocytes.

Immunofluorescence staining for TRPC1 in WT murine chondrocytes before and following bFGF stimulation demonstrated the relocation of TRPC1 channels from the ER to the cell surface, where they are expected to contribute to the influx of Ca^2+^ (Figure 1F) (22). In cartilage tissue, TRPC1 protein expression was examined using IHC staining of paraffin sections of human knee articular cartilage from healthy and OA donors. While expression was evident in chondrocytes throughout the cartilage in samples from healthy joints, protein levels were reduced significantly between early stages and advanced stages of human OA disease (Figure 1, G and H). A similar phenomenon was observed in mice, where expression in WT murine knee joints was assessed in sham and DMM knees 2 weeks and 8 weeks following surgery. A reduced level of TRPC1 protein was found within the articular cartilage at both early and later stages of OA development (Figure 1, I and J).

By mining previously published datasets, we could show that *TRPC1* is downregulated at the gene expression level by human chondrocytes in vitro by IL-1β as an inflammatory stimulus (GSE75181) (Supplemental figure 1B) as well as in cartilage explants following mechanical injury (Supplemental figure 1C) (23).

### Trpc1 is required for articular cartilage homeostasis following DMM challenge.

**Figure 2:**
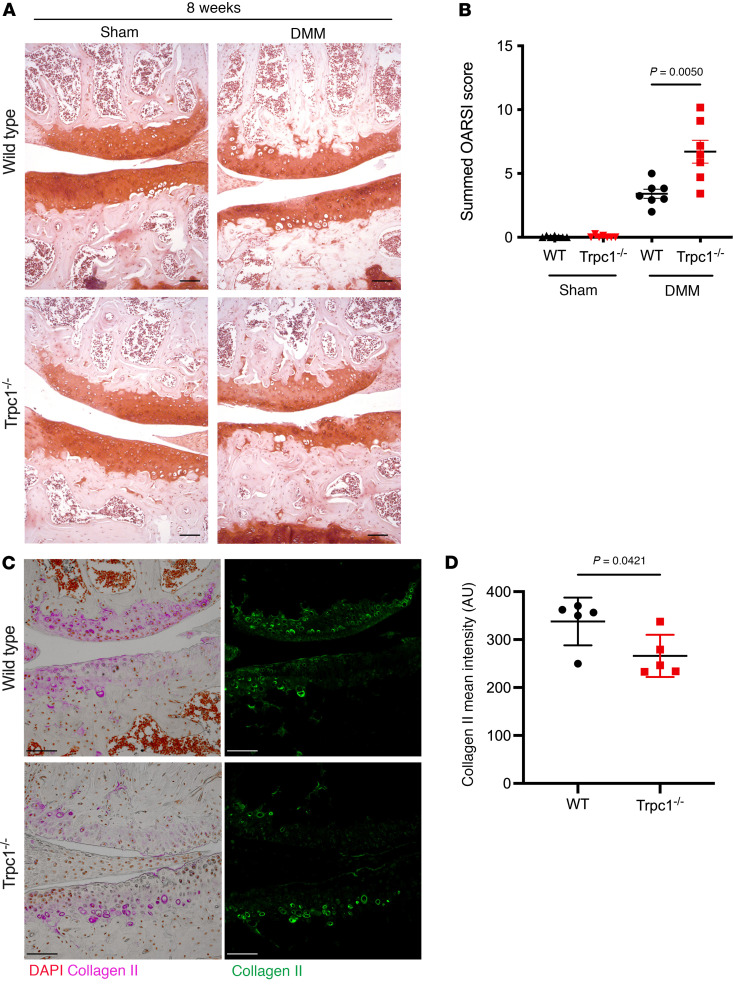
Assessment of articular cartilage phenotype 8 weeks after surgery (sham versus DMM). (**A**) Representative images of Safranin-O–stained paraffin sections of medial compartments of knee joints of WT control and *Trpc1^–/–^* mice 8 weeks after DMM. Scale bar: 100 μm. (**B**) Summed OARSI score of medial and lateral compartments of WT and Trpc1*^–/–^* knee joints 8 weeks after DMM (*n* = 7). (**C**) Immunofluorescence detection of collagen type II in the medial compartments of WT and *Trpc1^–/–^* knee joints 8 weeks after DMM. Scale bar: 100 μm. For isotype negative control staining, see Supplemental Figure 3D. (**D**) Quantification of the mean fluorescent signal intensity of collagen type II immunofluorescence within the remaining medial articular cartilage (*n* = 5). For all graphs, data are expressed as mean ± SEM and *P* values were determined by unpaired *t* test.

To investigate the possible effects of a lack of TRPC1 expression, the knee joint structure and cartilage phenotype of *Trpc1*-deficient mice were compared with WT controls. No developmental anomalies were found during examination of newborn murine knee joints (Supplemental Figure 3A and Supplemental Figure 5), while the body weights, articular cartilage thickness, and joint phenotype of unchallenged 10-week-old mice were found to be equal to controls (Supplemental Figure 3, B and C), indicating that the lack of TRPC1 does not lead to an altered phenotype during skeletal development. However, *Trpc1^–/–^* mice subjected to the DMM model of surgically induced joint instability were found to develop a more severe OA-like phenotype, as illustrated by an increased OARSI score assessment of cartilage defects 8 weeks following surgery (Figure 2, A and B). This phenotype was accompanied by a decreased level of type II collagen staining within the articular cartilage of *Trpc1^–/–^* joints after DMM, indicating an increased collagen matrix breakdown (Figure 2, C and D). These findings indicate that the absence of TRPC1 exacerbates cartilage degradation observed 8 weeks after DMM surgery.

### Early alterations in knee joint phenotype are evident in Trpc1^–/–^ mice 2 weeks after DMM.

**Figure 3:**
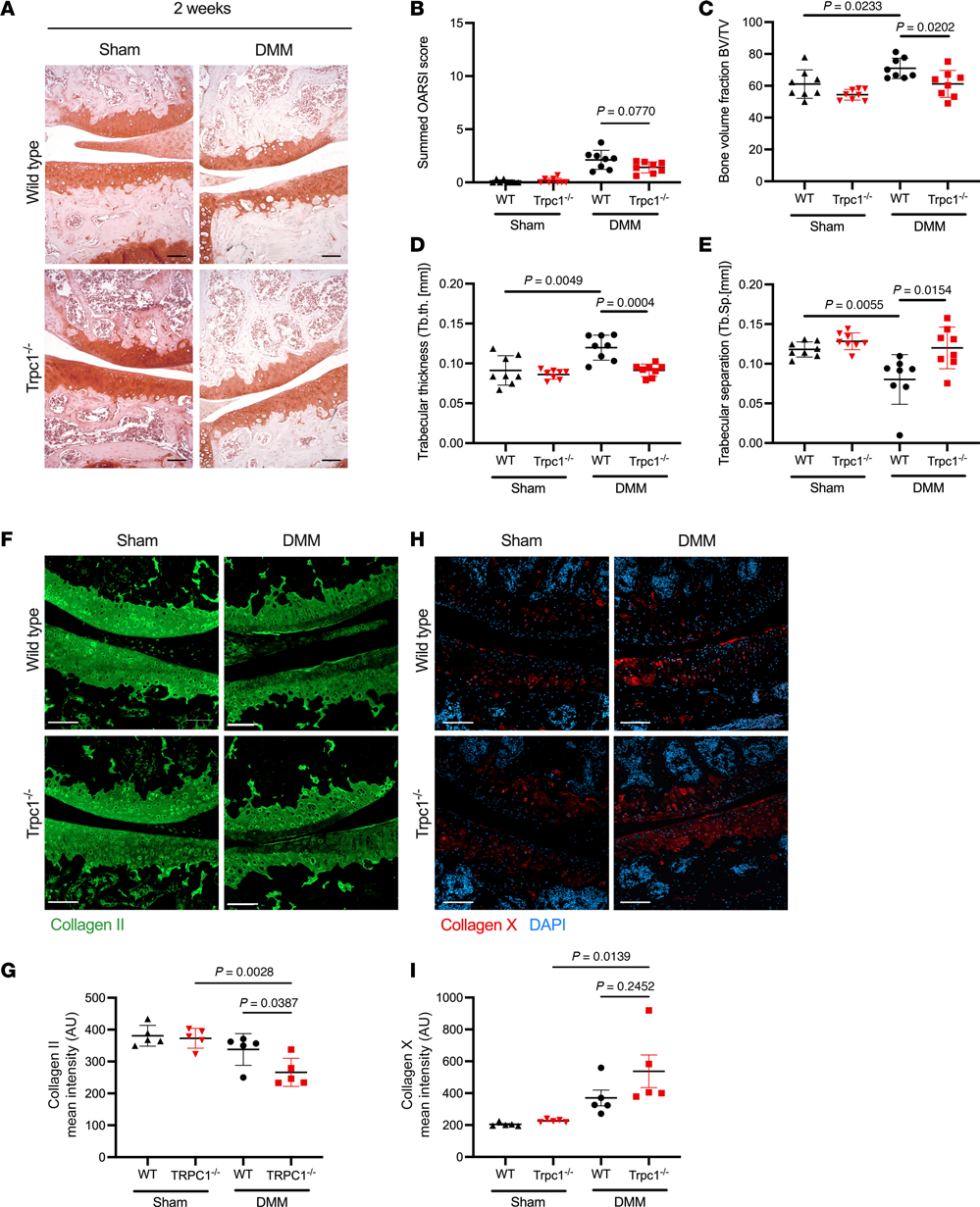
Assessment of early cartilage and subchondral bone changes 2 weeks after DMM. (**A**) Representative images of Safranin-O–stained paraffin sections of medial compartments of knee joints of WT control and *Trpc1^–/–^* mice 2 weeks after surgery (sham versus DMM). Scale bar: 100 μm. (**B**) Summed OARSI score of medial and lateral compartments of WT and *Trpc1^–/–^* knee joints 2 weeks after DMM (*n* = 8). (**C**) Bone volume fraction (normalized for total volume; BV/TV) fraction measurement of WT and *Trpc1^–/–^* knee joint tibial subchondral bone 2 weeks after sham control or DMM (*n* = 8). (**D**) Trabecular thickness (Tb.th. [mm]) measurement of WT and *Trpc1^–/–^* knee joint tibial subchondral bone 2 weeks after sham control or DMM surgery (*n* = 8). (**E**) Trabecular separation (Tb.Sp. [mm]) in WT and *Trpc1^–/–^* knee joint tibial subchondral bone 2 weeks after sham control or DMM (*n* = 8). (**F**) Immunofluorescence detection of collagen type II (green) in the medial compartments of WT and *Trpc1^–/–^* knee joints 2 weeks after DMM. Scale bar: 100 μm. For isotype negative control staining, see Supplemental Figure 3D. (**G**) Quantification of the mean fluorescent signal intensity of collagen type II immunofluorescence within the medial articular cartilage (*n* = 5). One-way ANOVA with Tukey’s multiple-comparison test. (**H**) Immunofluorescence detection of collagen type X (red) in the medial compartments of WT and *Trpc1^–/–^* knee joints 2 weeks after DMM. Scale bar: 100 μm. For isotype negative control staining, see Supplemental Figure 3E. (**I**) Quantification of the mean fluorescent signal intensity of collagen type X immunofluorescence within the medial articular cartilage (*n* = 5).One-way ANOVA with Tukey’s multiple-comparisons test. For all graphs, data are expressed as mean ± SEM. *P* values were determined by unpaired *t* test unless stated otherwise.

In order to analyze the role of TRPC1 during the early initiation phase of OA development, prior to significant structural damage to the articular surface, *Trpc1^–/–^* murine knee joints 2 weeks after DMM were compared with WT controls with histological analysis showing no obvious differences in articular cartilage structure (Figure 3, A and B). μCT analysis of the tibial plateau at this time point demonstrated that while WT controls begin to exhibit an increase in subchondral bone density including an increase in trabecular thickening, *Trpc1^–/–^* joints were slower to respond, if at all, to the alteration in joint biomechanics caused by the DMM (Figure 3, C–E, and Supplemental Figure 4). These more subtle changes were accompanied by a reduction in type II collagen (Figure 3, F and G), where a significant decrease in protein levels in DMM knees compared with sham controls was evident in *Trpc1^–/–^* murine knee articular cartilage but not in WT. Similarly, a greater increase in type X collagen as a marker of chondrocyte hypertrophic differentiation was found in *Trpc1^–/–^* tibial cartilage following DMM than in WT controls (Figure 3, H and I), indicating that, when triggered by joint destabilization, the lack of TRPC1 may accelerate the phenotypic switch of articular chondrocytes to a more pathological state that results in the more pronounced cartilage breakdown visibly observed by OARSI scoring at 8 weeks after DMM.

### Development of a cellular senescent phenotype in Trpc1^–/–^ articular cartilage following DMM.

**Figure 4:**
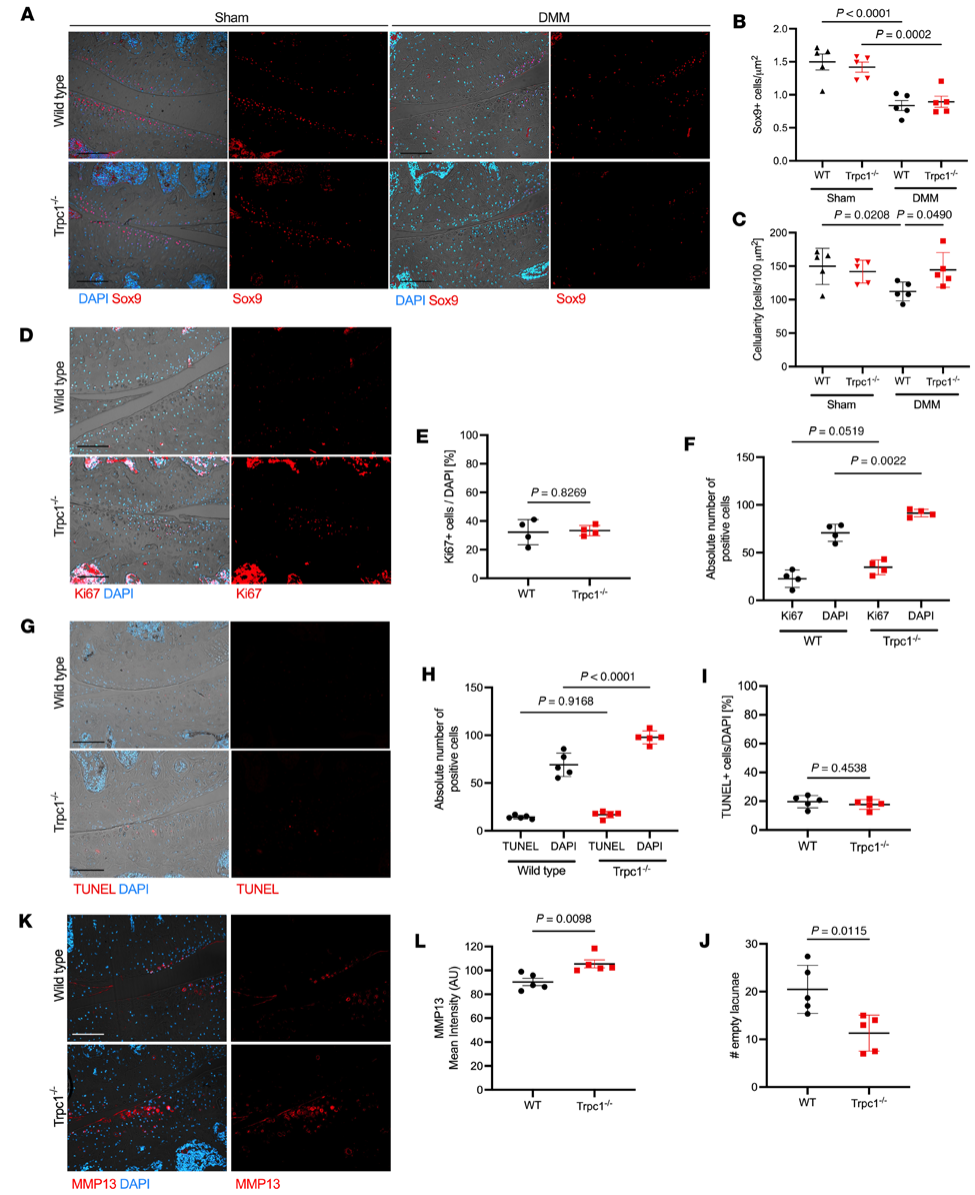
TRPC1-dependent phenotypic changes in cartilage during early stages of OA development in mice. (**A**) Immunofluorescence detection of Sox9 in the medial compartments of WT and *Trpc1^–/–^* knee joints 2 weeks after DMM. DAPI used as a nuclear counterstain. (**B**) Quantification of density of Sox9^+^ cells within the medial tibial articular cartilage. One-way ANOVA with Tukey’s multiple-comparison test. (*n* = 5). (**C**) Comparison of overall cellularity of medial tibial articular cartilage in WT and *Trpc1^–/–^* 2 week after DMM and sham mice as defined by number of DAPI^+^ cells per 100 μm^2^. One-way ANOVA with Tukey’s multiple-comparison test. (*n* = 5). (**D**) Immunofluorescence detection of Ki67 as a marker of cell proliferation in medial compartments of murine knee joints 2 weeks after DMM. DAPI used as a nuclear counterstain. (**E**) Quantification of number of Ki67^+^ cells within the medial tibial articular cartilage normalized for the total number of DAPI^+^ cells. Unpaired *t* test (*n* = 4). (**F**) Absolute quantification of the number of Ki67^+^ cells and number of DAPI^+^ cells within the medial tibial articular cartilage 2 weeks after DMM. One-way ANOVA with Tukey’s multiple-comparison test (*n* = 4). (**G**) TUNEL staining of medial compartments of murine knee joints 2 weeks after DMM. DAPI used as a nuclear counterstain. (**H**) Quantification of number of TUNEL^+^ cells normalized for total number of cells within the tibial articular cartilage. One-way ANOVA with Tukey’s multiple-comparison test (*n* = 5). (**I**) Absolute numbers of TUNEL^+^ and DAPI^+^ cells within the tibial articular cartilage 2 weeks after DMM. (**J**) Quantification of number of empty lacunae within the tibial articular cartilage 2 weeks after DMM. (**K**) Immunofluorescence detection of MMP13 in medial compartments of WT and *Trpc1^–/–^* knee joints 2 weeks after DMM. DAPI used as a nuclei counterstain. (**L**) Quantification of mean MMP13 staining intensity. Unpaired *t* test (*n* = 5) unless stated otherwise. Scale bars: 100 μm. For isotype negative control stainings, see Supplemental Figure 3.

In order to further understand the chondrocyte phenotypic changes occurring 2 weeks after DMM in the *Trpc1^–/–^* animals, cartilage sections were next assessed for Sox9 expression, a key promotor of chondrocyte differentiation (24). A comparable decline of Sox9^+^ cells was observed in the cartilage of both WT and *Trpc1*-deficient animals 2 weeks after DMM compared with corresponding sham animals (Figure 4, A and B). Remarkably, however, while the absolute number of Sox9^+^ cells was reduced, the overall number of surviving DAPI^+^ cells within the same area was found to be significantly higher in *Trpc1^–/–^* cartilage than in that of WT mice following DMM (Figure 4C), indicating that a large number of cells no longer expressing Sox9 remain within the articular cartilage of *Trpc1^–/–^* mice following OA induction. Next, Ki67 staining was used to assess whether the increased cellularity in *Trpc1^–/–^* cartilage reflected a higher proliferative rate of the remaining cells; however, although the absolute number of Ki67^+^ proliferating cells was higher in *Trpc1^–/–^* cartilage than in WT, this increase was normalized by the overall increase in DAPI^+^ cellularity (Figure 4, D–F), meaning that no overall increase was observed in the proliferation activity of the remaining cells at this time point. Similarly, although an assessment of active cell death using TUNEL staining demonstrated more TUNEL^+^ cells within *Trpc1^–/–^* knee articular cartilage after DMM, this difference was lost when normalized to the total number of cells remaining in the cartilage (Figure 4, G–I). Interestingly, an evaluation of the number of empty lacunae left within the tibial cartilage of WT and *Trpc1^–/–^* mice 2 weeks after DMM suggested that a lower rate of cell death may have occurred in *Trpc1^–/–^* mice at an earlier time point (Figure 4J), which may account for a higher overall cellularity at this stage. Notably, immunofluorescence staining demonstrated an increased expression of MMP13 within the articular cartilage of *Trpc1^–/–^* mice 2 weeks after DMM (Figure 4, K and L). These data suggest that TRPC1 may be necessary to regulate the survival rate of dedifferentiated chondrocytes during OA development and triggers the question of what role these additional remaining cells might be playing.

### Expression profiles of murine cartilage and subchondral bone are altered in Trpc1^–/–^ mice 2 weeks after DMM.

**Figure 5:**
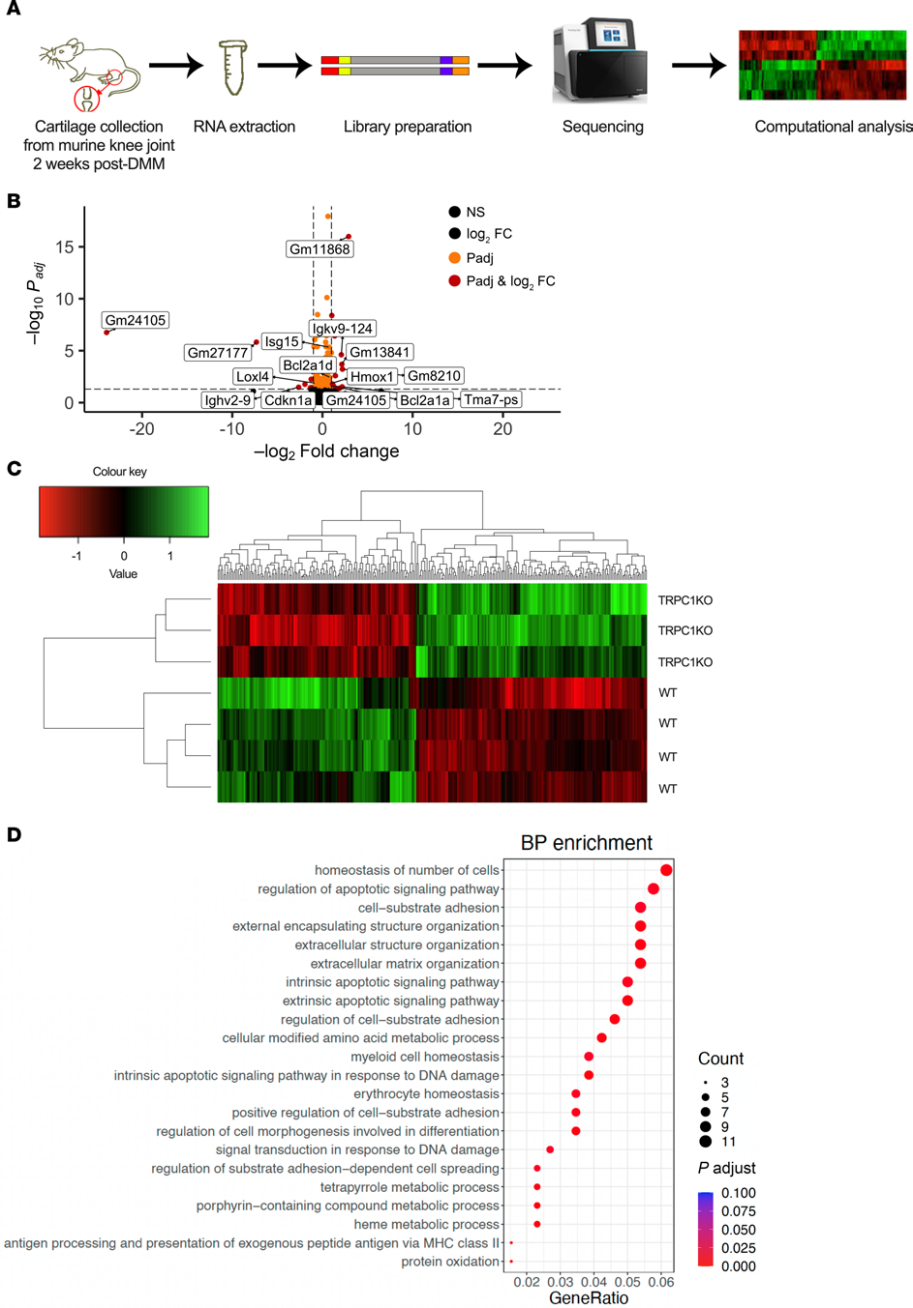
RNA expression profiles show an altered response to DMM in cartilage and subchondral bone. (**A**) Diagram of RNA-Seq workflow. (**B**) Volcano plot of cartilage gene expression fold-changes (FC, log_2_ scale) in *Trpc1^–/–^* relative to WT mice 2 weeks after DMM and corresponding FDR-adjusted *P* values (*P*_adj_, –log_10_ scale). Dashed lines delineate cut-off values (Benjamini-Hochberg FDR, 0.05; FC, 1.5), and red dots highlight significantly differentially expressed genes (DEGs) (i.e., FC > 1.5 with *P*_adj_ < 0.05). (**C**) Heatmap showing DEG profiles (genes with FC > 1.5) in the individual samples from both *Trpc1^–/–^* and WT groups, where green represents upregulation and red represents downregulation. (**D**) Biological pathway (BP) enrichment dot plot where gene ratio on the *x* axis represents the ratio of the total DEGs in the given GO term on the *y* axis. The size of each dot symbolizes the number of DEGs annotated with a specific term.

To gain further insight into the early pathogenic mechanisms causing the effects observed during OA development in the joints of *Trpc1^–/–^* animals, RNA-Seq was performed on femoral condyle and tibial plateau cartilage collected 2 weeks after DMM from WT and *Trpc1^–/–^* mice (Figure 5A). Up- and downregulated expression of all genes was first visualized on a volcano plot (Figure 5B). In total, 294 significantly differentially expressed genes (DEGs), defined as logFC > 1.5 and *P*a_dj_ < 0.05, were identified. The top 100 DEGs can be viewed within Supplemental Table 1. Next, hierarchical clustering of all genes with logFC > 1.5 helped visualize expression variation among animals and highlight the consistent DMM-driven transcription profile that emerged in *Trpc1^–/–^* mice (Figure 5C). Gene ontology (GO) enrichment analysis identified the biological processes (BP) with the greatest distinctions between *Trpc1^–/–^* and WT animals (Figure 5D). Interestingly, and perhaps not surprisingly based on the findings presented so far, the top 6 enriched BP that emerged in *Trpc1^–/–^* animals were: homeostasis of number of cells (GO:0048872); regulation of apoptotic signaling pathway (GO:2001233); cell-substrate adhesion (GO:0031589); and organization of ECM and extracellular structures (GO:0043062, GO:0030198, GO:0031589).

### TRPC1 deficiency leads to chondrocyte phenotypic instability and an increased susceptibility to cellular senescence.

**Figure 6:**
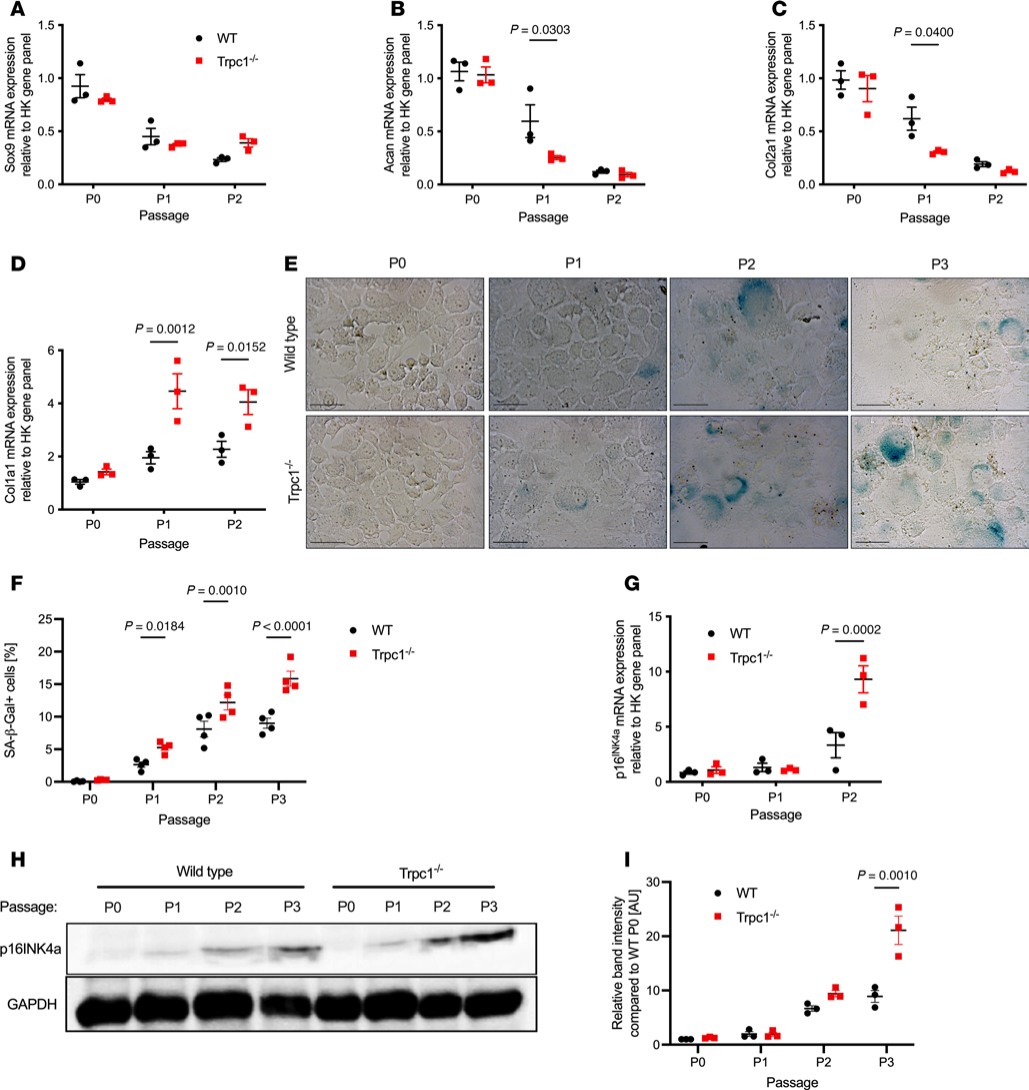
In vitro analysis of the effect of *Trpc1* deficiency upon chondrocyte phenotypic stability. (**A**–**D**) qPCR analysis of *sox9*, aggrecan, *col2a1*, and *col10a1* gene expression in murine WT and *Trpc1^–/–^* chondrocytes during serial in vitro passage. Two-way ANOVA with Tukey’s multiple-comparison test (*n* = 3). (**E**) Representative images of SA–β-Gal staining (blue) of WT and *Trpc1^–/–^* chondrocytes at equivalent confluence, visible by DIC counterimage, during serial passage. Scale bar: 50 μm. (**F**) Quantification of number of SA–β-Gal^+^ cells normalized for total number of cells during WT and *Trpc1^–/–^* chondrocytes during serial passage. Two-way ANOVA with multiple comparisons (*n* = 4, 20 images per condition). (**G**) qPCR analysis of *p16INK4a* gene expression in WT and *Trpc1^–/–^* chondrocytes during serial passage. Two-way ANOVA with multiple comparisons (*n* = 3). (**H**) Western blot of p16^INK4a^ protein levels in WT and *Trpc1^–/–^* chondrocytes during serial passage. GAPDH used as a loading control. (**I**) Quantification of Western blotting for p16^INK4a^ in WT and *Trpc1^–/–^* chondrocytes during serial passage. Two-way ANOVA with multiple comparisons (*n* = 3).

Given the differences shown in the RNA-Seq in the expression of genes related to cell homeostasis and ECM organization following DMM, we next examined the effect of the absence of TRPC1 on the phenotype of articular chondrocytes during in vitro passage. Key phenotypic genes were expressed at comparable high levels by freshly expanded chondrocytes taken from 4- to 6-day-old WT and *Trpc1^–/–^* unchallenged knee joints (Figure 6, A–D); however, during serial passage, which trials the ability of chondrocytes to maintain their phenotypic stability (25), a number of differences are apparent. Sox9 gene expression remained approximately equal between genotypes during passage (Figure 6A). However, the expression of aggrecan and Col2a1 were significantly lower during passage in *Trpc1^–/–^* chondrocytes as compared with WT controls (Figure 6, B and C). Conversely, Col1a1 expression was significantly higher during passage of *Trpc1^–/–^* chondrocytes compared with controls (Figure 6D), indicating an accelerated dedifferentiation of the chondrocytes.

Cellular plasticity and senescence are complexly interlinked processes (26). To measure whether the accelerated dedifferentiation observed in *Trpc1^–/–^* chondrocytes was associated with the development of an altered senescent phenotype, WT and *Trpc1^–/–^* chondrocytes were stained for β-galactosidase activity, an indicator of cellular senescence. Very little staining was observed in either genotype at P0, but an increased proportion of Senescence-associated–β-Galactosidase^+^ (SA–β-Gal^+^) cells was observed during passage in *Trpc1^–/–^* chondrocytes compared with controls, indicating an accelerated differentiation toward a senescent fate (Figure 6, E and F). Similarly, p16^INK4a^, another marker of cellular senescence, was found to be expressed at significantly higher levels in *Trpc1^–/–^* chondrocytes during passage than in controls at both RNA (Figure 6G) and at protein (Figure 6, H and I) levels. Taken together, these findings indicate that, in the absence of TRPC1, chondrocytes exhibit an accelerated differentiation toward a senescent fate in vitro.

**Figure 7:**
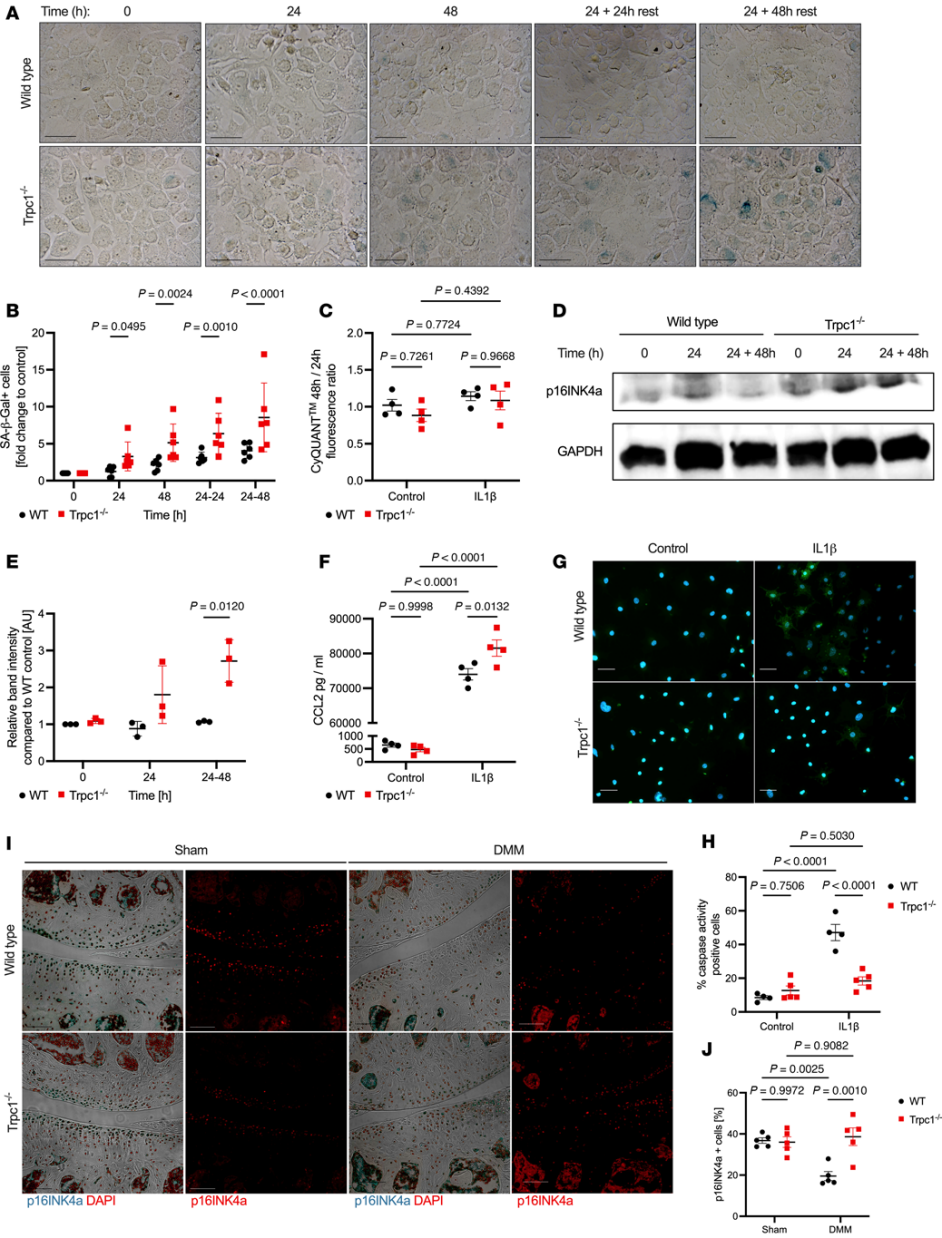
TRPC1 is required for protection against cellular senescence driven by IL-1β and during murine OA development. (**A**) Representative images of SA–β-Gal stainings (blue) of P0 WT and *Trpc1^–/–^* chondrocytes treated with 10 ng/mL IL-1β for either 24 hours, 48 hours, or for 24 hours followed by an additional 24 or 48 hours culture in control medium (rest). (**B**) Quantification of number of SA–β-Gal^+^ cells normalized to those in control untreated samples (*n* = 5). (**C**) CyQUANT fluorescence quantification of WT and *Trpc1^–/–^* chondrocytes measured at 24 and 48 hours after IL-1β stimulation to give a ratio representing proliferation rate (*n* = 4). (**D**) Western blot of p16^INK4a^ protein levels in WT and *Trpc1^–/–^* chondrocytes treated with 10 ng/mL IL-1β for either 24 hours or for 24 hours followed by 48 hours culture in control medium. (**E**) Quantification of Western blotting for p16^INK4a^ in WT and *Trpc1^–/–^* chondrocytes treated with 10 ng/mL IL-1β for either 24 hours or for 24 hours followed by 48 hours culture in control medium (*n* = 3). (**F**) CCL-2 concentration of control and IL-1β–treated chondrocyte supernatants measured by ELISA (*n* = 4). (**G**) Representative images of cleaved caspase activity in chondrocytes treated with IL-1β for 24 hours plus 48 hours rest. (**H**) Quantification of cleaved caspase activity in WT and *Trpc1^–/–^* chondrocytes following IL-1β stimulation (WT, *n* = 4; *Trpc1^–/–^*, *n* = 5). (**I**) Immunofluorescence detection of p16^INK4a^ in medial compartments of WT and *Trpc1^–/–^* murine knee joints 2 weeks after DMM. DAPI used as a nuclear counterstain. Scale bar: 100 μm. For isotype negative control staining, see Supplemental Figure 3I. (**J**) Quantification of number of p16^INK4a+^ cells within the medial articular cartilage normalized for the total number of DAPI^+^ cells (*n* = 5). Two-way ANOVA with multiple comparisons.

Finally, we wanted to determine if TRPC1 deficiency influences whether chondrocytes shift toward a senescent phenotype following exposure to IL-1β as an inflammatory stimulus associated with OA disease pathogenesis (27, 28). Treatment with IL-1β led to an increase in the proportion of SA–β-Gal^+^ cells with a significantly higher rate of detection found in *Trpc1^–/–^* chondrocytes compared with WT (Figure 7, A and B). This effect was exacerbated when observed following further culture in IL-1β–free control medium for an additional 24 or 48 hours, indicating that once triggered, the inflammation-driven development of cellular senescence in *Trpc1^–/–^* chondrocytes may become chronic even without the presence of IL-1β. No differences were detected between the proliferation rates of *Trpc1^–/–^* chondrocytes cultured either in control or IL-1β–treated conditions (Figure 7C). At the protein level, a significantly higher amount of p16^INK4a^ was detected in *Trpc1^–/–^* chondrocytes compared with WT controls following IL-1β stimulation (Figure 7, D and E), further indicating the development of a more senescence-like phenotype of *Trpc1^–/–^* cells following IL-1β exposure.

We then tested whether the *Trpc1^–/–^* chondrocytes exhibited any of the secretory phenotype characteristic of senescent cells. Interestingly, the SASP CCL-2 concentration was found to be significantly higher within the supernatant of IL-1β–treated *Trpc1^–/–^* chondrocytes as compared with that of WT controls (Figure 7F), although this was not the case for other SASPs, including IL-6 and MMP3 (Supplemental Figure 6, A and B). In order to understand the mechanism behind the increased cellularity observed in the *Trpc1^–/–^* osteoarthritic cartilage, we tested whether TRPC1 regulation of Ca^2+^ signaling, which is strictly required for caspase activity via cytochrome C (29), is required for caspase-mediated apoptosis. Following exposure to IL-1β, *Trpc1^–/–^* chondrocytes were unable to upregulate caspase activity, indicating a failure to initiate and undergo apoptosis (Figure 7, G and H).

These observations also left us with the question of whether chondrocyte senescence was altered in *Trpc1^–/–^* animals after DMM. Immunofluorescence staining analysis demonstrated that, although the proportion of p16^INK4a^-positive chondrocytes were comparable between WT controls and *Trpc1^–/–^* following sham surgery, 2 weeks after DMM, the proportion of p16^INK4a^-positive cells in *Trpc1^–/–^* cartilage was significantly higher (Figure 7, I and J), again indicating an increased contribution of cellular senescence to the accelerated OA phenotype of *Trpc1^–/–^* mice after DMM.

## Discussion

OA is characterized by the progressive destruction of the articular cartilage accompanied by degenerative changes in surrounding joint tissues. However, we currently are not able to fully understand the mechanistic stages linking early alterations in joint mechanics, inflammation, and cellular metabolism to the phenotypic changes typical of chondrocytes during OA that have been demonstrated to drive tissue breakdown.

TRPC1 sits within the ER membrane and functions within the Ca^2+^-shuttling mechanism required by cells to refill the intracellular calcium stores that are emptied repeatedly during activation of both biochemical signaling and mechanically induced pathways. We have hypothesized that the cyclic nature of normal ambulatory movement, involving repeated loading and unloading events that induce shear and compressive stresses upon chondrocytes within the articular cartilage, require TRPC1 activity to sustain the refilling of intracellular calcium stores that are repeatedly cleared during stimulation. We have been able to demonstrate that TRPC1 is depleted during the early phases of OA development and that this loss of TRPC1 leads to failure of murine articular cartilage to maintain its homeostasis during the mechanical challenge provided by the DMM model.

While initial expectations were that *TRPC1* could be downregulated at the gene expression level following early inflammatory or mechanical stimulation as seen in previous ex vivo human datasets (23, 30), microarray data from previous murine studies do not indicate that *Trpc1* expression is modulated in this way within our examined species and time points (31, 32) (publicly available dataset GSE169077), which aligns with lack of downregulation at the gene expression level in patients with OA (GSE114007) (19). Instead, we can hypothesize that the reduction in TRPC1 protein levels evident in immunohistology analyses of both murine and human cartilage during OA development may occur due to degradation or removal of the channels. Indeed, evidence of proteolytic degradation of the related TPRC5 and TRPC6 channels by calpain (33, 34), which is found within synovial fluid of patients with OA (35), exists alongside the identification of TRPC1 as a substrate of caspase-11 within the control of NLRP3 inflammasome activation (36).

This study demonstrates the consequences of TRPC1 loss from chondrocytes, most clearly shown within the DMM model where genetic deletion resulted in an increased severity of OA-like cartilage degeneration 8 weeks following instability induction. Although the use of a chondrocyte-specific conditional mutant line would additionally eliminate any indirect effects of *Trpc1* deletion related to metabolic changes or systemic immune cell influence, no alterations in overall body weight, joint structure, or cartilage structure were evident in either our newborn or age-matched unchallenged mice, suggesting that TRPC1 plays no significant systemic role in either skeletal development or joint maturation. While other evidence from studies in skeletal muscle also show that sustained and repeated stimulation, in the form of muscle contraction during forced exercise, leads to functional failure as a consequence of an inability to refill intracellular stores sufficiently (18), our examination of cells obtained from *Trpc1*-deficient mice demonstrate that TRPC1 is required specifically in chondrocytes for the maintenance of phenotypic stability, the maintained expression of type II collagen and aggrecan, and for protection against the development of a pathogenic senescent phenotype.

While significant phenotypic changes to chondrocytes are expected following DMM induction, *Trpc1^–/–^* mice exhibited an array of differential gene expression compared with WT controls, as displayed in the RNA-Seq analysis. These data support our immunostaining analyses that demonstrate that, at a time point of 2 weeks after DMM, while an increased number of cells remain within the articular cartilage, they express markers of cellular senescence including p16^INK4a^ and MMP13, display markers of irregular cell number homeostasis, and have a low expression of stable chondrocyte markers. Our RNA-Seq DEGs do not significantly overlap with the senescence transcriptomic signature identified by Saul et al. (37). This may firstly reflect the use in our study of a posttraumatic rather than an aging OA model, but it also illustrates the incomplete senescence secretory phenotype exhibited by the *Trpc1^–/–^* chondrocytes. Similarly, other expected examples of senescence markers such as Cdkn1a were not found to be upregulated in our RNA-Seq, potentially as a consequence of our choice of time point for analysis. Our in vitro investigations into the effects of *Trpc1* deficiency on IL-1β–induced cellular challenge demonstrate that, while the increase in expression of specific SASPs such as CCL-2 is evident, this partial senescent phenotype is exacerbated by the failure of *Trpc1^–/–^* chondrocytes to undergo Ca^2+^-mediated caspase–driven apoptosis. We see that *Trpc1^–/–^* cells, under conditions of challenge, fail to activate the physiological pathway leading to apoptosis and rather acquire a senescent-like “zombie” phenotype, whereby they are unable to perform other homeostatic functions such as respond to FGF signaling (21). Previous studies have demonstrated the important role of SASPs in skin wound healing and in liver regeneration (38, 39), suggesting that the lack of a full SASP phenotype in *Trpc1^–/–^* mice after DMM might actually be detrimental to any repair attempt within the cartilage. These factors likely contribute to the accelerated development of cartilage breakdown, which results in the more severe phenotype in *Trpc1*-deficient mice that we observe 8 weeks after DMM.

The accumulation of senescent cells during OA has been shown to contribute to joint degradation via the secretion of proinflammatory factors including IL-6, IL-17, and IL-1β known as the senescence-associated secretory profile (SASP) (40). Senolytic approaches aiming at removing these cells from cartilage have been shown to improve outcome within animal models of OA (41). Although not yet successful within so-far limited human OA trials, the therapeutic targeting of senescence development, removal of senescent cells, or inhibition of SASP-related elements represent key opportunities in the search for DMOADs (42). The RNA-Seq data collected within this study reveal the transcriptional activity linking the disruption to the normal chondrocyte phenotype caused by the failure of chondrocytes to sufficiently maintain homeostatic calcium signaling to the eventual breakdown of the articular cartilage. These mechanisms may become targets for future translational studies aimed at limiting the effects of the loss of TRPC1 during OA development.

Mechanosensitive ion channels have been previously implicated within cartilage biology, where deletion of TRPV4 was observed to decrease OA-related changes during aging (15), while PIEZO-1 and PIEZO-2 have roles in response to injury (17), with inflammation driving an increase in PIEZO-1 that increases both basal and responsive intracellular calcium concentrations to provide a feed-forward loop within OA pathogenesis (43). Other channels that are demonstrated to participate within the signaling pathways of known homeostatic mechanisms, such as within CXCR2 signaling (44, 45), may also represent valuable druggable targets for phenotypic modulation of chondrocytes; however, the role of TRPC1 and its loss during OA development gives us several strategies for therapeutic development that require evaluation. While agonists able to specifically activate this channel only are not yet available, we believe that the activation step may be secondary to the need to preserve, reexpress, and stabilize TRPC1 within the ER or to facilitate its translocation to the cell surface.

## Methods

### Sex as a biological variable.

This study examined human cartilage tissue from both male and female patients, with no differences found between sexes. The murine study exclusively used male mice because male mice exhibited less variability in phenotype.

### Human cartilage samples.

Samples of knee cartilage were collected during implantation of total knee arthroplasty as well as in unicondylar joint replacement at the Department of Orthopaedic Surgery of the University Hospital Magdeburg (Magdeburg, Germany). The patients/participants provided their written informed consent to participate in this study. Details of the included patients can be found in Supplemental Table 2. OA severity was determined histologically using the OARSI score. Knee cartilage from patients who had died without a history of OA was used as a control. The absence of OA was assessed histologically.

### Mice.

Trpc1^tm1Lbi^ mice within the C57BL/6J background, originally generated by Dietrich et al. (46), were used for all in vitro and in vivo TRPC1-KO experiments. This mouse lacks exon 8, which sits within the transmembrane region of the channel, leading to a premature stop codon within the *Trpc1* transcript. All mice were housed within ventilated cages in a 12-hour day/night cycle with food and water ad libitum.

DMM was carried out as described previously (3, 47) on 10-week-old male *Trpc1^–/–^* mice and age- and sex-matched WT controls from within the same C57BL/6J breeding colony. Right knees were subjected to DMM, while a sham surgery was performed on the left knee of each mouse, where the skin and joint capsule were opened but no damage was caused to the cartilage or ligaments. Mice were euthanized at either 2 weeks or 8 weeks after DMM, and knee joints were collected for histological processing or RNA library construction.

### Histology and immunostaining.

Human and murine tissues were fixed in 4% paraformaldehyde overnight, dehydrated, embedded in paraffin, and cut in 5 μm sections. Safranin-O staining (0.2%, pH 4.2) was used for histological analysis of the articular cartilage. Stained sections were imaged using an Axiovert Z1 microscope (Zeiss), and OA severity was assessed using the OARSI scoring system (48). Between 3 and 5 sections per samples spanning each human sample, or weight-bearing area of the murine knee joint, were scored by 2 independent scorers blinded to the experimental group.

For immunostaining of TRPC1 and of phenotypic markers, deparaffinized sections were rehydrated and subjected to pepsin (1,100 U/mL in 0.02 % HCl) digestion for 45 minutes at 37°C for antigen retrieval. Tissues were blocked in 20% fetal calf serum (FCS) in PBS with 0.2% Tween20 for 1 hour at room temperature. Sections were stained with antibodies as listed in Supplemental Table 3 and detected either via fluorescence microscopy or by DAB detection (Vector Labs) and light microscopy using an Axiovert Z1 microscope (Zeiss) after mounting using Mowiol (Sigma-Aldrich) on glass slides. Quantification was performed as stated in individual figures, either by the counting of individual positive cells normalized for area of interest using ImageJ (NIH; v.2.1), or by quantification of positive staining intensity using ImageJ. Analyses were conducted on between 3 and 5 individual sections per joint for each staining, with the mean value per animal used for statistical comparison. Only the articular cartilage of the tibial plateau and femoral condyles are included within analyses, as outlined within Figure 1I. Meniscus, subchondral bone, developing osteophytes, and bone marrow are excluded.

### Chondrocyte cell culture and stimulation.

Primary articular chondrocytes were isolated from the knee joints of 4- to 6-day-old mice as described previously (49) using 1 mg/mL Collagenase IV in complete medium — DMEM high glucose (Thermo Fisher Scientific) containing 10% FCS superior (Biochrom GmbH), 1% sodium pyruvate (Thermo Fisher Scientific) and 1% penicillin/streptomycin (Sigma-Aldrich). Chondrocytes were used at 80% confluency for experiments within the first passage unless otherwise stated and stimulated with 10 ng/mL of murine IL-1β (Preprotech) where required.

### Immunofluorescence staining of chondrocytes.

Immunofluorescence staining for TRPC1 was performed to detect the localization of the channel within the cells. Chondrocytes were grown to 80% confluence on glass coverslips, fixed in 4% PFA for 10 minutes, and washed twice with PBS. Cells were permeabilized with PBS–Triton X for 10 minutes and quenched with 0.025% ammonium chloride in PBS for two 5-minute washes. After blocking with 20% FCS in PBS for 1 hour at room temperature, cells were incubated with primary and secondary antibodies as listed in Supplemental Table 3 and detected using fluorescence microscopy using an Axio Imager 2.0 with Apotome (Zeiss).

### Immunoblotting.

Cell lysates containing equal amounts of protein and loading dye were separated by electrophoresis on 10% SDS page gels (Bio-Rad) alongside PageRuler Plus prestained protein ladder (Thermo Fisher Scientific) at 80 V. Proteins were transferred to a nitrocellulose membrane using a Trans-blot Turbo system (Bio-Rad), blocked in BSA (5% in TBS-T) at room temperature for 3 hours, and incubated overnight at 4°C with the primary antibody as listed in Supplemental Table 3; they were then washed 3 times in TBS-T and incubated with HRP-linked secondary antibody for 1 hour at room temperature. Membranes were washed, incubated with ECL solution, and visualized via chemiluminescence using a Fusion FX Western blot imager (Vilber Lourmat).

### TUNEL staining.

Cells undergoing cell death were detected using the In Situ Cell Death Detection Kit, TMR red (Merck), according to the manufacturer’s instructions. In brief, paraffin sections were deparaffinized, rehydrated, and permeabilized. Sections were incubated with the TMR red label for broken DNA strands and counterstained using DAPI. After mounting in Mowiol. Staining was visualized using an Axiovert Z1 microscope (Zeiss). Sections pretreated with DNase I (1 U) for 10 minutes were used as a positive control.

### Quantitative PCR (qPCR).

Total RNA was extracted from chondrocyte cultures using TRIzol (Invitrogen) as previously described using chloroform separation, isopropanol, and 70% ethanol washes. cDNA was synthesized using the TaqMan Reverse Transcription kit (Invitrogen) according to the manufacturers’ instructions. In total, 1,000 ng of RNA was transcribed using oligo(dT)16-primers, and relative ΔCT gene expression levels were analyzed using primer sequences or TaqMan probes (Thermo Fisher Scientific) listed in Supplemental Table 4 using a CFX384 qPCR cycler (Bio-Rad) and β-2-microglobulin (B2m), β-actin (Actb) and glyceraldehyde 3-phosphate dehydrogenase (Gapdh) forming a housekeeping (HK) control gene panel.

### RNA-Seq.

Total RNA was extracted from femoral head and tibial cartilage and from subchondral bone pooled from 2–3 mouse knees per sample (DMM-WT, *n* = 3; DMM-TRPC1, *n* = 4), was assessed for quality using an RNA Nano chip on a Bioanalyzer (Agilent). Average RNA integrity number (RIN) for the 7 samples was 7.9 ± 1.7 SD. Samples were quantified using the Qubit RNA BR fluorometric assay (Thermo Fisher Scientific) on the DS-11 spectrophotometer (Denovix). In total, 300 ng total RNA of each sample was used to prepare sequencing libraries using the TruSeq Stranded Total RNA with RiboZero as per manufacturer’s recommendations (Illumina) and as previously described (50). Library quality was assessed on a Bioanalyzer high-sensitivity DNA chip. Average library size was 300 ± 9 bp SD. Seven libraries were volumetrically pooled and sequenced on an Illumina NextSeq 550 instrument for 75 paired-end read cycles at the Schroeder Arthritis Institute (Krembil).

Demultiplexing of samples was performed using bcl2fastq conversion tool (v2.19.1.403). Quality assessment of each sample revealed high-quality of reads by Phred score (90.4% > Q30; 80 ± 14 million reads/per end). To maintain a minimum read length of 25 bp after trimming of adapters, Cutadapt (v2.5) software was executed (~3% reads being contaminated) along with trimming of data. Splice-aware alignment of reads using a Hierarchical Graph FM (HGFM) index method was performed using HISAT2 software (v2.1.0 with parameters --rna-strandness RF –dta) against mouse reference genome (GRCm38). To populate the abundance of transcripts based on the reference genome and transcriptome, StringTie (v2.0.3 with parameters -e -B -G referencetranscripts.gtf) was run to generate output as table format files. These files were processed using the script “prepDE.py” from StringTie software to collect the raw gene expression levels for downstream processing.

Raw gene expression levels consisted of 55,401 genes measured across the 7 samples. After filtering for lowly expressed genes, defined as genes with < 2 samples with a count per million ≤ 1, a total of 16,429 genes remained for further analysis. Changes in RNA-Seq expression were evaluated using a method that assumes a negative binomial distribution (51). Differential expression analyses were performed using function DESeq (default parameters) in R package DESeq2 (52). Pairwise comparisons between groups of interest were calculated and tested for statistical differences in expression levels, visualized using a volcano plot created in R. The FDR correction was used to adjust *P* values (53), where 5% identified statistically significant DEGs. All analyses were performed in R (v3.5.0). DEGs were further represented using heatmap.2 function in gplots (v3.0.1). GO BP enrichment analysis was performed using clusterProfiler (v4.0.5 in R 4.1.0), and resulting plots were generated in R using ggplot (v2 3.3.5).

### Intracellular calcium measurement.

To measure the levels of intracellular Ca^2+^ mobilization, chondrocytes were seeded within a 96-well plate (30,000 cells/well) and loaded with Fluo-4 reagent before stimulation according to the manufacturer’s instructions. Cells were then monitored for fluorescence signal using the Axiowert A1 with live cell imaging CO_2_ chamber and heating plate (Zeiss) under standard culture conditions. Basal levels were measured via fluorescence imaging using an excitation wavelength of 494 nm and emission wavelength of 516 nm, every 15 seconds for 2 minutes under resting conditions, before any stimulus was added. Following stimulation using a standardized 5 μL volume of either PBS, ionomycin, thapsigargin or bFGF, further measurements were taken every 15 seconds for 5 minutes. The change in fluorescence intensity was calculated relative to basal levels.

### SA–β-Gal detection.

β-Gal activity, a marker of cells undergoing senescence, was detected by cytochemical cleavage of the chromogenic substrate 5-bromo-4-chloro-3-indoyl β-D-galactopyranoside (X-Gal) into an insoluble compound at the pH of 6.0. Cells were fixed with glutaraldehyde/formaldehyde (0.2%/2% in PBS), washed in PBS, and covered with the X-Gal staining solution (54) for 20 hours at 37°C. Cells were then washed with methanol and air dried before imaging using an Axiovert Z1 microscope (Zeiss).

### ELISA.

Protein concentrations within supernatant — collected following 24 hours of incubation with 10 ng/mL IL-1β followed by 48 hours further culture in control medium — were collected and used for ELISA quantification with the following kits according to the manufacturer’s instructions: Mouse CCL2/JE/MCP-1 Quantikine ELISA (R&D Systems), Mouse IL-6 Quantikine ELISA (R&D Systems), and Mouse Total MMP-3 Quantikine ELISA (R&D Systems). Quantification was performed with reference to the standard solutions provided in each kit.

### Proliferation assay.

Chondrocyte proliferation was measured in vitro using the CyQUANT Cell Proliferation Assay (Thermo Fisher Scientific) according to the manufacturer’s instructions, which uses a fluorescent nucleic acid dye to measure the DNA content within a sample. The proliferation rate was calculated by dividing the content measured after 48 hours of incubation with either control or 10 ng/mL IL-1β with that measured after 24 hours of incubation.

### Caspase activity assay.

Caspase activity as a measure of caspase-mediated apoptosis was measured using the Generic Caspase Activity Assay Kit (Fluorometric – Green) (Abcam). Chondrocytes incubated with either control or 10 ng/mL IL-1β–containing media for 24 hours followed by 48 hours of further culture in control medium were labeled according to the manufacturer’s instructions, before fixation in 4% paraformaldehyde and visualization using an Axiovert Z1 microscope (Zeiss).

### μCT imaging and analysis.

μCT scanning of knee joints was performed using a Skyscan 1176 scanner (Bruker), with a 0.5 aluminium filter, 0.3° rotation with 10 images per step. Scans were reconstituted using NRecon (version 1.7.4.6), and images were positioned for quantitative analysis using DataViewer (Version 1.5.6.3) software. The tibial plateau was selected and analyzed using CTAn (Version 1.1.8.9.0) software, where subchondral bone plate and subchondral bone trabecular parameters could be separated. Bone parameters analyzed included subchondral bone thickness, bone volume fraction, trabecular thickness, and trabecular separation.

### Statistics.

Statistical analyses were performed using GraphPad Prism version (v9.0) software. Individual tests and statistical significance levels are indicated within figures and figure legends. Unless stated otherwise, data are presented as mean ± SEM, and *P* < 0.05 was considered statistically significant. Two-tailed Student’s *t* test or 2-way ANOVA with Tukey’s multiple-comparison test was used for statistical analysis unless otherwise stated in the legend.

### Study approval.

For human studies, written informed consent was received prior to sample donation. The study was reviewed and approved by the IRB of the Medical School, Otto-von-Guericke University, Magdeburg (no 28/20). All animal experiments were approved by the local ethics committee Landesamt für Natür, Umwelt und Verbraucherschutz Nordrhein-Westfalen (LANUV, 84.02.04.2017.A050 and 84-02.05.50.15.005) and were carried out in compliance with ARRIVE guidelines.

### Data availability.

All data relevant to the study are included in the article or uploaded as supplemental information, aside from RNA-Seq data that will be available in a public open access repository upon acceptance of the manuscript. Supporting data values associated with the main manuscript and supplemental material are available within the Supporting Data Values file.

## Author contributions

MS and SL contributed experimental design, data acquisition, data analysis and interpretation, writing of the manuscript. OEG, P Potla, and CP contributed data analysis and interpretation. JB contributed provision of human tissue samples. SB, LW, SK, P Paruzel, RA, AT, and JI contributed data acquisition. FDA contributed data analysis and interpretation as well as writing of the manuscript. MK contributed experimental design, data analysis and interpretation, and writing of the manuscript. TP contributed conceptualization, experimental design, data analysis and interpretation, and writing of the manuscript. JS contributed conceptualization, data acquisition, data analysis and interpretation, and writing of the manuscript. All authors have edited and approved the manuscript.

## Supplementary Material

Supplemental data

Unedited blot and gel images

Supporting data values

## Version 1 12/24/2024

In-Press Preview

## Version 2 02/10/2025

Electronic publication
