# Supplementary material for: TRPC1 links calcium signaling to cellular senescence in the protection against posttraumatic osteoarthritis
Source: JCI Insight. 2024 Dec 24;10(3):e182103. doi: 10.1172/jci.insight.182103 (PMC11948585; doi:10.1172/jci.insight.182103)
Supplement: Supplemental data [file jciinsight-10-182103-s205.pdf]

Transient receptor potential channel 1 (TRPC1) links intracellular calcium signaling to cellular senescence and is required for protection against post-traumatic osteoarthritis.

Supplemental materials

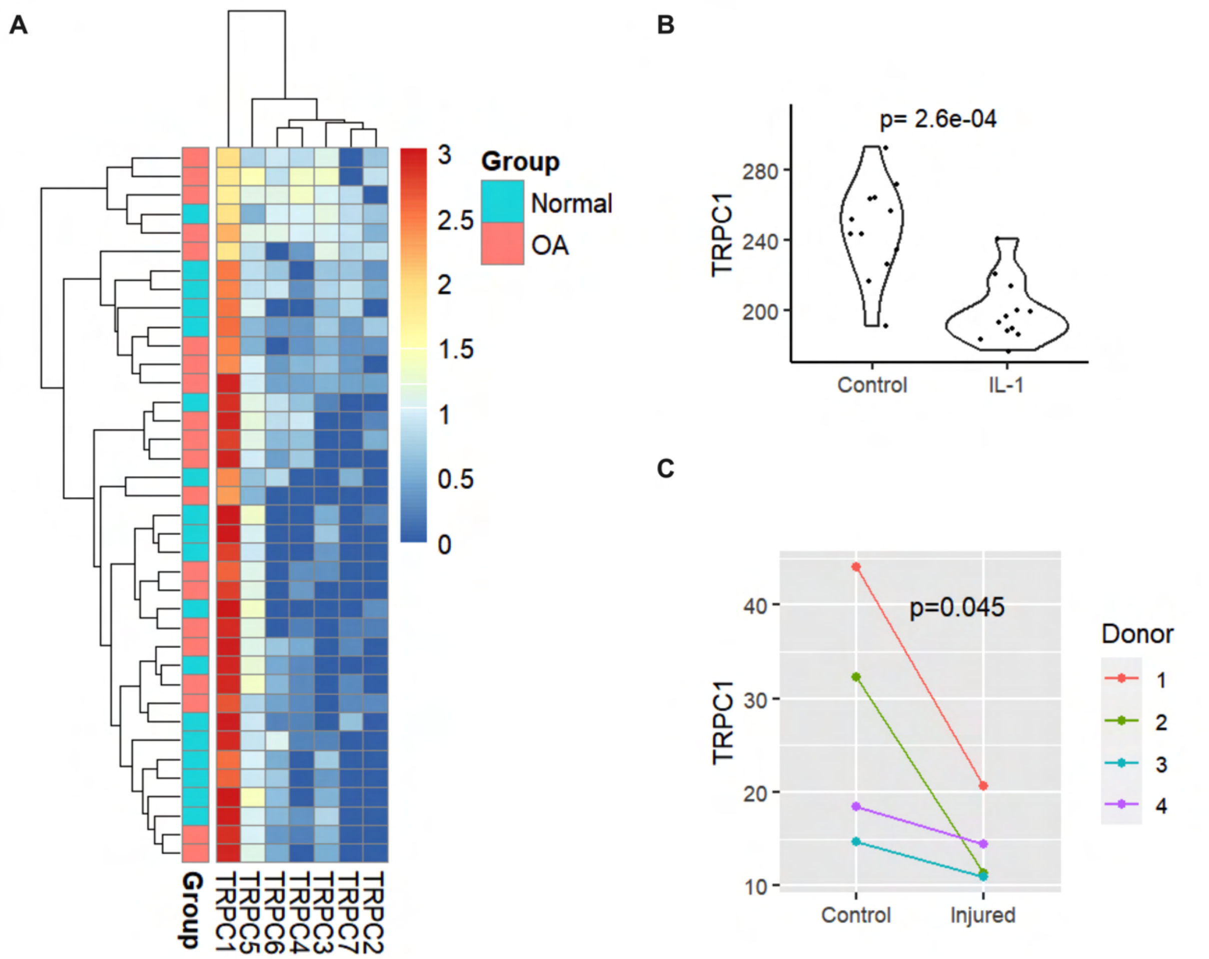

Supplemental Figure 1. (A) Post-hoc analysis of RNAseq data for TRPC channel expression in human normal and OA cartilage (GEO: GSE114007) (1) (B) Post-hoc analysis of microarray data from IL-1β treated human OA chondrocytes showing downregulation of TRPC1 gene expression following IL-1β application (GEO: GSE75181) (2). (C) Microarray gene expression data comparing TRPC1 expression in human cartilage before and following cutting injury (3) P-value: paired t-test after reciprocal transformation (n = 4).

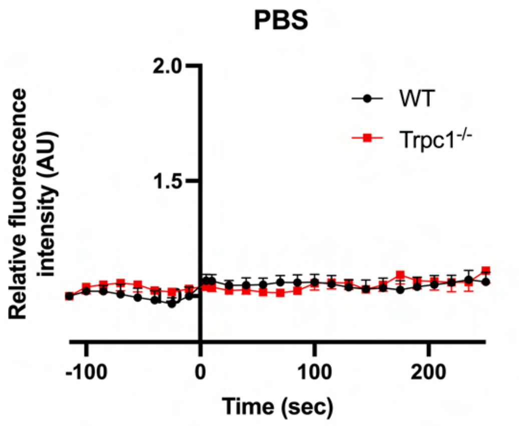

Supplemental Figure 2. Time course analysis of intracellular Ca^{2+} levels as measured by fluorescence intensity of loaded Fluo-4 Ca^{2+} indicator in wild type and $Trpc1^{-/-}$ chondrocytes during stimulation with PBS (negative control)

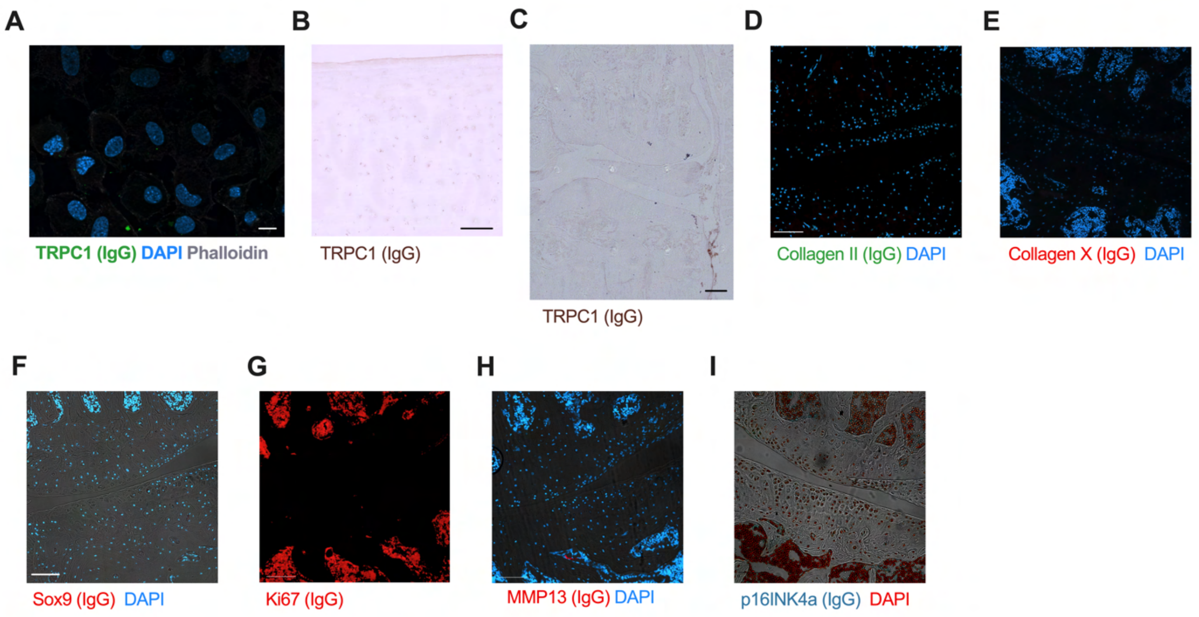

Supplemental Figure 3. (A) IgG isotype negative control for TRPC1 immunofluorescence staining on P0 murine chondrocytes. Scale bar: 20 µm. (B) IgG isotype negative control for TRPC1 immunohistochemical staining on human healthy cartilage paraffin section. Scale bar: 100 µm. (C) IgG isotype negative control for TRPC1 immunohistochemical staining on murine sham operated knee joint section. Scale bar: 100 µm. (D) IgG isotype negative control for type II collagen immunofluorescence staining on murine sham operated knee joint section. Scale bar: 100 µm. (E) IgG isotype negative control for type X collagen immunofluorescence staining on murine 2 weeks post-DMM knee joint section. Scale bar: 100 µm. (F) IgG isotype negative control for Sox9 immunofluorescence staining on murine sham operated knee joint section. Scale bar: 100 µm. (G) IgG isotype negative control for Ki67 immunofluorescence staining on murine 2 weeks post-DMM knee joint section. Scale bar: 100 µm. (H) IgG isotype negative control for MMP-13 immunofluorescence staining on murine 2 weeks post-DMM knee joint section. Scale bar: 100 µm. (I) IgG isotype negative control for p16INK4a immunofluorescence staining on murine 2 weeks post-DMM knee joint section. Scale bar: 100 µm. Where present, DAPI (blue) was used to label nuclei.

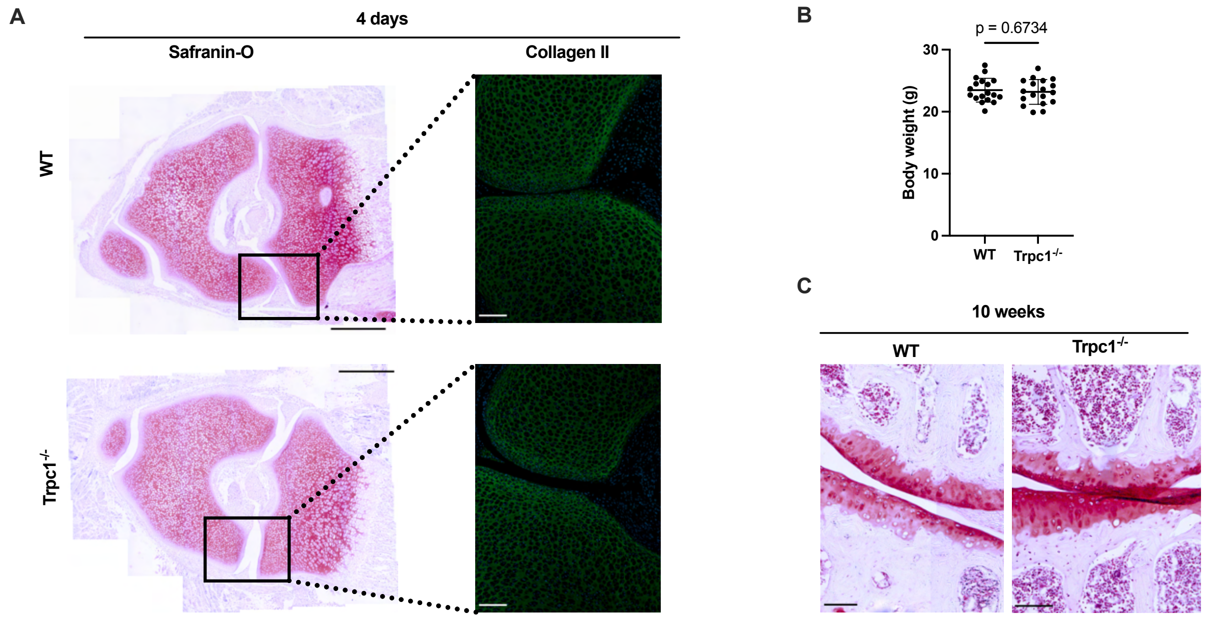

Supplemental Figure 4. (A) Safranin-O histology and type II collagen immunofluorescence staining of murine 4 day old wild type and *Trpc1*$^{-/-}$ knee joint sections demonstrating no obvious abnormalities caused by Trpc1 deficiency during development. Scale bars: 500 µm (Safranin-O) and 100 µm (type II collagen, green). (B) Comparison of total body weights of 10 weeks old male wild type and *Trpc1*$^{-/-}$ mice at time of DMM. P value: unpaired t-test (n = 18). (C) Safranin-o staining of 10-week old unchallenged male murine knee joint sections. Scale bar: 100 µm.

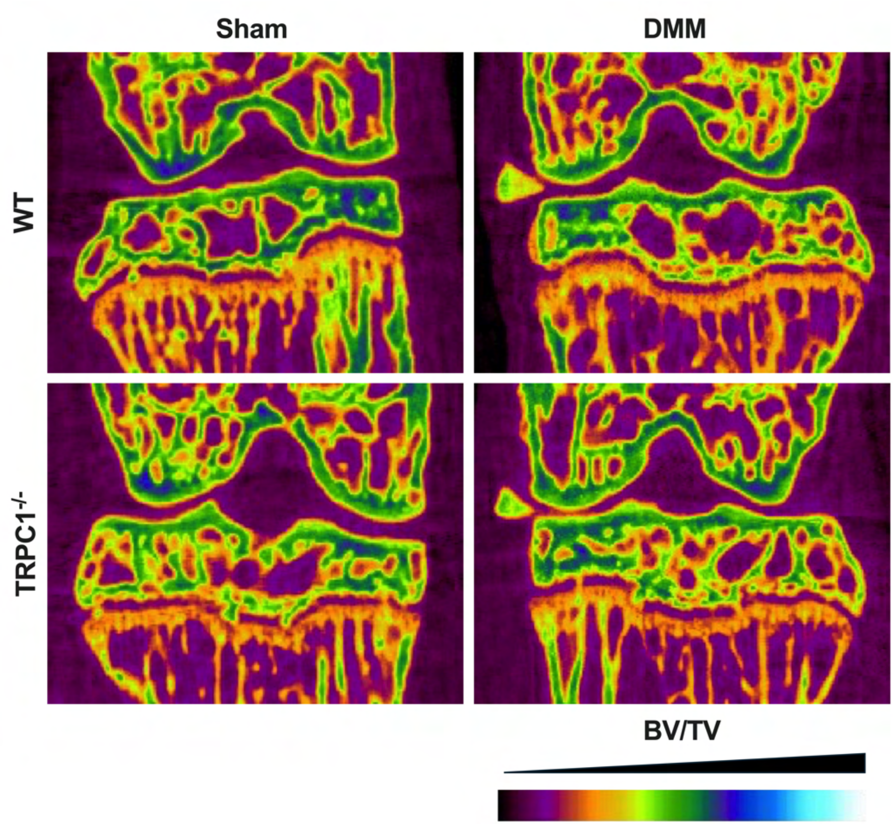

Supplemental Figure 5. Representative frontal cross section images taken from microCT analyses of wild type and *Trpc1*$^{-/-}$ knee joints 2 weeks post-DMM. Colour spectrum illustrates changes in BV/TV.

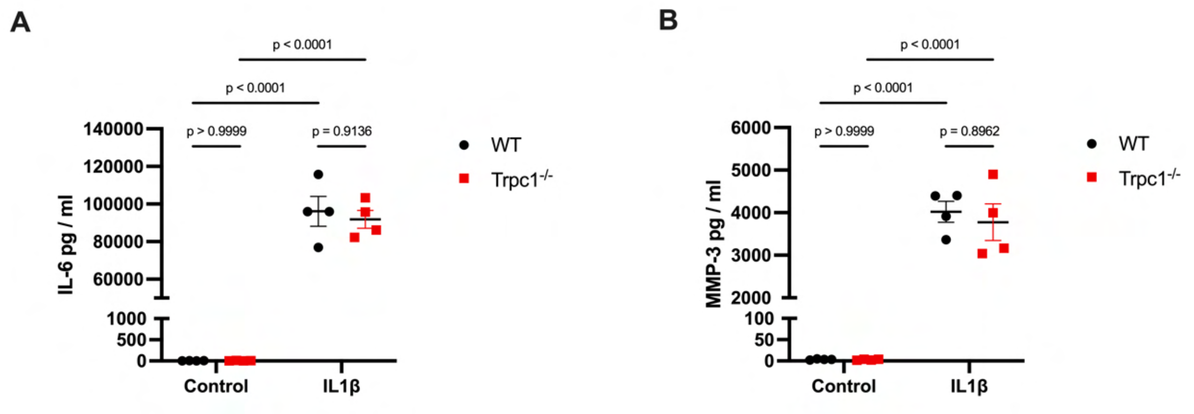

Supplemental Figure 6. Protein concentration analyses of murine IL-6 and MMP-3 within supernatants of wild type and *Trpc1*$^{-/-}$ chondrocytes after 24 hours stimulation with 10 ng/ml IL-1β and 48 hours further culture in control medium measured by ELISA (n = 4). P values: 2-way ANOVA with multiple comparisons.

Supplemental Table 1. Top 100 differentially expressed genes found within RNA sequencing comparison articular cartilage collected from wild type and *Trpc1*$^{-/-}$ mice 2 weeks post-DMM.

gene_id	geneSymbol	baseMean	log2FoldChange	lfcSE	stat	pvalue	padj
ENSMUSG00000050550	Gm11868	654,54	2,90671818	0,3121	9,3119	1,255E-20	1,0302E-16
ENSMUSG00000094497	Gm8210	1133,71	2,216069522	0,4597	4,8212	1,427E-06	0,00060075
ENSMUSG00000083833	Gm13841	1202,01	2,153237441	0,4226	5,0949	3,489E-07	0,00020456
ENSMUSG00000096632	Igkv9-124	161,49	2,091116993	0,3786	5,5236	3,322E-08	2,479E-05
ENSMUSG00000096712	Gm15454	204,10	1,535250393	0,2301	6,6726	2,513E-11	6,8747E-08
ENSMUSG00000096715	Igkv3-4	143,83	1,43307761	0,3245	4,4161	1,005E-05	0,002578
ENSMUSG00000094546	Ighv1-26	754,58	1,362296619	0,2149	6,3397	2,302E-10	3,7795E-07
ENSMUSG00000116637	Gm8130	79,63	1,230868486	0,2595	4,7432	2,104E-06	0,00082225
ENSMUSG00000116875	Morf4l1-ps1	258,74	1,041319953	0,1467	7,0997	1,251E-12	4,106E-09
ENSMUSG00000094446	Gm6344	356,97	0,991965094	0,1766	5,6175	1,938E-08	1,5801E-05
ENSMUSG00000062611	Rps3a2	4418,44	0,958697538	0,1786	5,3682	7,953E-08	5,2225E-05
ENSMUSG00000058126	Tpm3-rs7	3720,32	0,922204706	0,1909	4,8304	1,362E-06	0,00058858
ENSMUSG00000099974	Bcl2a1d	129,08	0,831627005	0,1933	4,3028	1,686E-05	0,00369128
ENSMUSG00000035692	Isg15	1243,57	0,790102163	0,1355	5,8315	5,492E-09	4,7453E-06
ENSMUSG00000066361	Serpina3c	403,62	0,746323368	0,1578	4,7292	2,254E-06	0,00084341
ENSMUSG00000038871	Bpgm	24042,67	0,719360606	0,1665	4,3194	1,565E-05	0,00347116
ENSMUSG00000036594	H2-Aa	10198,99	0,627216499	0,1138	5,5103	3,583E-08	2,5576E-05
ENSMUSG00000044468	Tent5c	47083,11	0,61527824	0,0625	9,8451	7,193E-23	1,1809E-18
ENSMUSG00000090113	Nhlrc4	373,58	0,600002707	0,1305	4,5985	4,256E-06	0,00124781
ENSMUSG00000060586	H2-Eb1	5694,52	0,590545487	0,1053	5,6102	2,021E-08	1,5801E-05
ENSMUSG00000020641	Rsad2	24839,68	0,57672145	0,1317	4,3795	1,19E-05	0,00278983
ENSMUSG00000022748	Cmss1	367,51	0,56726312	0,1200	4,7286	2,26E-06	0,00084341
ENSMUSG00000081992	Gm13408	985,20	0,553932197	0,1298	4,2666	1,984E-05	0,00408861
ENSMUSG00000040569	Slc26a7	841,57	0,544000441	0,1238	4,3939	1,113E-05	0,00269637
ENSMUSG00000073421	H2-Ab1	6743,88	0,532691776	0,1220	4,3658	1,267E-05	0,00292853
ENSMUSG00000026348	Acmsd	595,91	0,515277168	0,1222	4,2168	2,478E-05	0,00478686
ENSMUSG00000034127	Tspan8	1926,78	0,501010638	0,1073	4,6675	3,049E-06	0,0010309
ENSMUSG00000032411	Tfdp2	21571,66	0,497572187	0,0647	7,6953	1,411E-14	7,7219E-11
ENSMUSG00000022051	Bnip3l	22669,13	0,495603975	0,1160	4,2720	1,937E-05	0,00407664
ENSMUSG00000078139	AK157302	1509,73	0,485021183	0,1038	4,6723	2,979E-06	0,0010309
ENSMUSG00000044792	Isca1	11732,58	0,481712056	0,0952	5,0622	4,144E-07	0,00022678
ENSMUSG00000040435	Ppp1r15a	10355,53	0,476060159	0,1138	4,1840	2,864E-05	0,00528342
ENSMUSG00000035472	Slc25a21	1277,46	0,469971541	0,0797	5,8975	3,69E-09	4,1602E-06
ENSMUSG00000042770	Hebp1	2974,00	0,466525391	0,1062	4,3932	1,117E-05	0,00269637
ENSMUSG00000028906	Epb41	55559,35	0,450217808	0,0767	5,8722	4,299E-09	4,1602E-06
ENSMUSG00000011179	Odc1	22000,69	0,446787085	0,1003	4,4529	8,474E-06	0,00220813
ENSMUSG00000028124	Gclm	9047,81	0,444875212	0,0700	6,3560	2,071E-10	3,7783E-07
ENSMUSG00000023926	Rhag	7621,03	0,439430617	0,0865	5,0784	3,806E-07	0,00021547
ENSMUSG00000090946	Ccdc71l	4615,42	0,40475105	0,0968	4,1801	2,914E-05	0,00531572

ENSMUSG00000027322	Siglec1	2895,53	0,399967298	0,0863	4,6320	3,621E-06	0,0011009
ENSMUSG00000024588	Fech	34158,88	0,39649454	0,0732	5,4138	6,172E-08	4,2216E-05
ENSMUSG00000040675	Mthfd1l	3352,66	0,387897236	0,0862	4,5022	6,726E-06	0,0017811
ENSMUSG00000029802	Abcg2	5581,07	0,384118904	0,0654	5,8715	4,319E-09	4,1602E-06
ENSMUSG00000042066	Tmcc2	32119,83	0,374662968	0,0854	4,3863	1,153E-05	0,00274291
ENSMUSG00000042225	Ammecr1	3543,00	0,358534661	0,0589	6,0821	1,186E-09	1,4978E-06
ENSMUSG00000006574	Slc4a1	107387,75	0,343172096	0,0818	4,1970	2,704E-05	0,00504513
ENSMUSG00000028436	Dcaf12	14430,42	0,33953874	0,0729	4,6546	3,246E-06	0,0010309
ENSMUSG00000027115	Kif18a	4817,40	0,338442336	0,0738	4,5838	4,567E-06	0,0013154
ENSMUSG00000028587	Orc1	1859,93	0,33362834	0,0694	4,8040	1,555E-06	0,00063822
ENSMUSG00000024726	Carnmt1	2297,05	0,327255356	0,0662	4,9462	7,567E-07	0,00037644
ENSMUSG00000026311	Asb1	9027,54	0,326437974	0,0723	4,5160	6,301E-06	0,00172405
ENSMUSG00000024014	Pim1	5470,22	0,323694296	0,0758	4,2730	1,929E-05	0,00407664
ENSMUSG00000041147	Brca2	6849,03	0,315660183	0,0680	4,6417	3,455E-06	0,00107015
ENSMUSG00000037458	Azin1	22638,39	0,313299149	0,0713	4,3964	1,101E-05	0,00269637
ENSMUSG00000014956	Ppp1cb	29803,94	0,311409223	0,0591	5,2715	1,353E-07	8,5437E-05
ENSMUSG00000042029	Ncapg2	13627,50	0,306160104	0,0663	4,6146	3,939E-06	0,00117582
ENSMUSG00000078652	Psme3	11512,23	0,294962666	0,0645	4,5732	4,803E-06	0,00135938
ENSMUSG00000044763	Trmt10c	1609,37	0,285364572	0,0630	4,5317	5,852E-06	0,00162836
ENSMUSG00000022100	Xpo7	46144,25	0,283446098	0,0581	4,8791	1,065E-06	0,00051446
ENSMUSG00000020124	Usp15	19152,63	0,266303671	0,0646	4,1214	3,765E-05	0,00650402
ENSMUSG00000018983	E2f2	16239,36	0,251422501	0,0578	4,3521	1,348E-05	0,00307457
ENSMUSG00000027452	Acss1	4400,92	0,234692666	0,0570	4,1191	3,803E-05	0,00650402
ENSMUSG00000002222	Rmnd5a	12160,35	0,176302913	0,0431	4,0889	4,334E-05	0,00711561
ENSMUSG00000064147	Rab44	9871,82	-0,274455612	0,0650	-4,2238	2,402E-05	0,00469484
ENSMUSG00000062031	Pgghg	1793,80	-0,291288317	0,0709	-4,1108	3,943E-05	0,00667388
ENSMUSG00000001248	Gramd1a	5759,92	-0,338672236	0,0727	-4,6567	3,214E-06	0,0010309
ENSMUSG00000026335	Pam	18845,73	-0,366101523	0,0865	-4,2321	2,315E-05	0,00457892
ENSMUSG00000028041	Adam15	3528,81	-0,367155974	0,0789	-4,6534	3,265E-06	0,0010309
ENSMUSG00000019970	Sgk1	5005,05	-0,375885954	0,0918	-4,0942	4,236E-05	0,00709679
ENSMUSG00000038168	P3h2	1500,45	-0,425216887	0,1029	-4,1326	3,587E-05	0,00626479
ENSMUSG00000040964	Arhgef10l	2422,98	-0,436914994	0,0903	-4,8368	1,319E-06	0,00058536
ENSMUSG00000024529	Lox	12761,01	-0,485552744	0,1002	-4,8454	1,264E-06	0,00057632
ENSMUSG00000018906	P4ha2	1956,33	-0,530675282	0,1298	-4,0890	4,333E-05	0,00711561
ENSMUSG00000070407	Hs3st3b1	563,22	-0,534602048	0,1074	-4,9781	6,42E-07	0,00032936
ENSMUSG00000082286	Pisd-ps1	1200,78	-0,542514183	0,0758	-7,1580	8,188E-13	3,3604E-09
ENSMUSG00000027848	Olfml3	6558,00	-0,557740206	0,1236	-4,5125	6,406E-06	0,00172405
ENSMUSG00000006369	Fbln1	805,99	-0,5642298	0,0883	-6,3875	1,686E-10	3,4596E-07
ENSMUSG00000024247	Pkdcc	1194,26	-0,582029945	0,0993	-5,8624	4,561E-09	4,1602E-06
ENSMUSG00000036412	Arsi	588,12	-0,606909589	0,1416	-4,2855	1,823E-05	0,00393819
ENSMUSG00000022780	Meltf	3915,07	-0,610547105	0,1255	-4,8630	1,156E-06	0,00054222
ENSMUSG00000023249	Parp3	1750,57	-0,619348953	0,1473	-4,2040	2,622E-05	0,00500555
ENSMUSG00000023800	Tiam2	3401,10	-0,621360012	0,1480	-4,1976	2,698E-05	0,00504513
ENSMUSG00000025504	Eps8l2	1417,50	-0,621590052	0,1463	-4,2496	2,141E-05	0,00434025
ENSMUSG00000056919	Cep162	1812,98	-0,630199781	0,1226	-5,1410	2,733E-07	0,00016621
ENSMUSG00000015709	Arnt2	382,37	-0,658474586	0,1582	-4,1610	3,168E-05	0,00565377

ENSMUSG00000054252	Fgfr3	3326,62	-0,663173742	0,1603	-4,1364	3,529E-05	0,00622878
ENSMUSG00000016200	Syt14	492,44	-0,678769167	0,1457	-4,6592	3,175E-06	0,0010309
ENSMUSG00000061718	Ppp1r1b	488,73	-0,700324759	0,1500	-4,6693	3,022E-06	0,0010309
ENSMUSG00000031952	Chst5	300,22	-0,710824866	0,1422	-4,9976	5,805E-07	0,00030742
ENSMUSG00000039084	Chad	13815,97	-0,737723134	0,1675	-4,4051	1,057E-05	0,00267026
ENSMUSG00000001494	Sost	2717,25	-0,739783454	0,1707	-4,3338	1,466E-05	0,00329594
ENSMUSG00000042073	Abhd14b	399,44	-0,742321486	0,1740	-4,2657	1,992E-05	0,00408861
ENSMUSG00000044006	Cilp2	3998,14	-0,787026677	0,1640	-4,7982	1,601E-06	0,0006412
ENSMUSG00000006403	Adamts4	350,19	-0,862034626	0,1392	-6,1943	5,855E-10	8,7376E-07
ENSMUSG00000032872	Cyb5r4	6144,96	-0,891695898	0,2102	-4,2421	2,215E-05	0,0044341
ENSMUSG00000042254	Cilp	40930,26	-0,937919323	0,1593	-5,8887	3,894E-09	4,1602E-06
ENSMUSG00000101429	BC055402	490,11	-1,067626246	0,2287	-4,6689	3,028E-06	0,0010309
ENSMUSG00000026077	Npas2	225,78	-1,248982415	0,3001	-4,1625	3,148E-05	0,00565377
ENSMUSG00000098975	Gm27177	363,36	-7,324938074	1,2034	-6,0870	1,151E-09	1,4978E-06
ENSMUSG00000105790	Gm24105	102,88	-23,94383664	3,6786	-6,5090	7,565E-11	1,7741E-07

Supplemental Table 2 Clinical parameters of patient donors providing cartilage samples within this study

Group	OARSI Score	Age (years)	Sex (m/f)	Synovitis (y/n)	BMI (kg/m^2)
Healthy	0	60 +/- 11.3	2/3	1/4	30.4 +/- 4.21
Mild	1.0 - 2.5	65 +/- 9.2	2/3	1/4	33.4 +/- 5.12
Advanced	> 3.0	69 +/- 9.9	5/3	1/7	26.2 +/- 4.30

Supplemental Table 3 List of antibodies used within this study

Antibody	Application	Manufacturer	Cat. No.	Conjugation
TRPC1	IHC-DAB	Alomone	a018	unconjugated
TRPC1	IF	Alomone	a010	unconjugated
Type II collagen	IHC-F	Merck	MAB8887	unconjugated
Type X collagen	IHC-F	Quartett	CO097-05	unconjugated
Sox9	IHC-F	Merck	MAB5535	unconjugated
Ki67	IHC-F	Leica Biosystems	Ki67P-CE	unconjugated
p16INK4a	WB	Abcam	ab211542	unconjugated
GAPDH	WB	Cell Signaling	5174	unconjugated
p16INK4a	IHC-F	Abcam	ab54210	unconjugated
Phalloidin	IF	ThermoFisher	AB22287	Alexa Fluor 647
Goat anti-rabbit IgG (H+L)	WB	DAKO	P0448	Horseradish peroxidase
Rabbit anti-mouse IgG (H+L)	IHC-F	ThermoFisher	A11059	Alexa Fluor 488
Donkey anti-mouse IgG (H+L)	IHC-F	ThermoFisher	A31570	Alexa Fluor 555
Donkey anti-rabbit IgG (H+L)	IHC-F	ThermoFisher	A10040	Alexa Fluor 546

Supplemental Table 4 List of primers using within this study

Gene	Taqman Probe	FW sequence	RV sequence
Actb	Mm02619580_g1	-	-
Aggrecan	Mm00545798_m1	-	-
B2M	Mm00437762_m1	-	-
Col1a1	Mm00801666_g1	-	-
Col2a1	Mm01309565_m1	-	-
Gapdh	Mm99999916_g1	-	-
Sox9	Mm00448840_m1	-	-
Actb	-	TGA CGG GGT CAC CCA CAC TGT GCC CAT CTA	CTA GAA GCA TTT GCG GTG GAC GAT GGA GG
B2M	-	AGA GGT CCT TTT CAC CAG CA	TCA GTC TCA GTG GGG GTG AA
Gapdh	-	AGC AAG GAC ACT GAG CAA GAG AGG	GGG TCT GGG ATG GAA ATT GTG AGG
p16INK4a	-	CCC AAC GCC CCG AAC T	GCA GAA GAG CTG CTA CGT GAA
