# Supplementary figures and images for: TRPC1 links calcium signaling to cellular senescence in the protection against posttraumatic osteoarthritis
Source: JCI Insight. 2024 Dec 24;10(3):e182103. doi: 10.1172/jci.insight.182103 (PMC11948585; doi:10.1172/jci.insight.182103)

Full unedited gel for Figure 6H

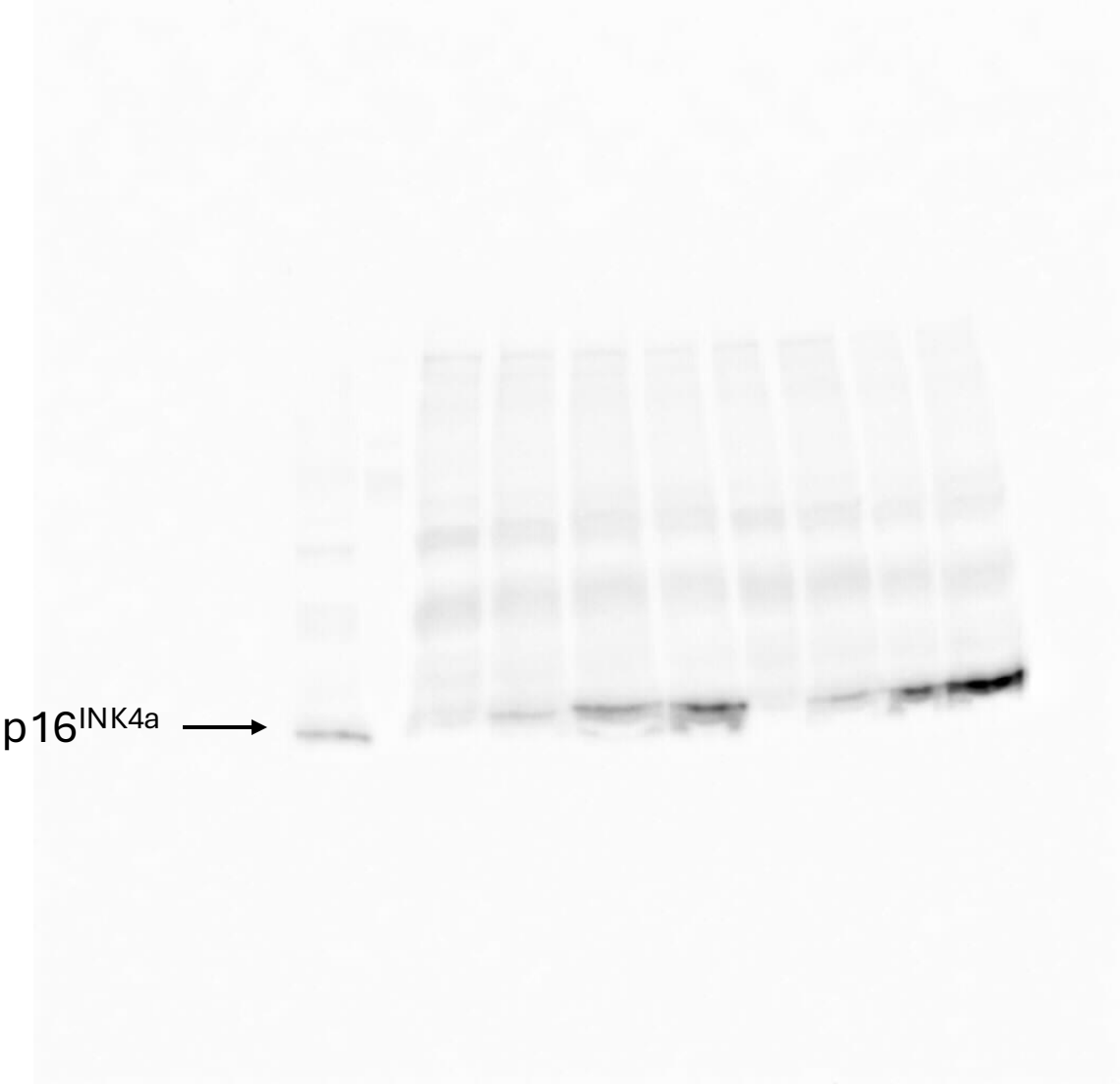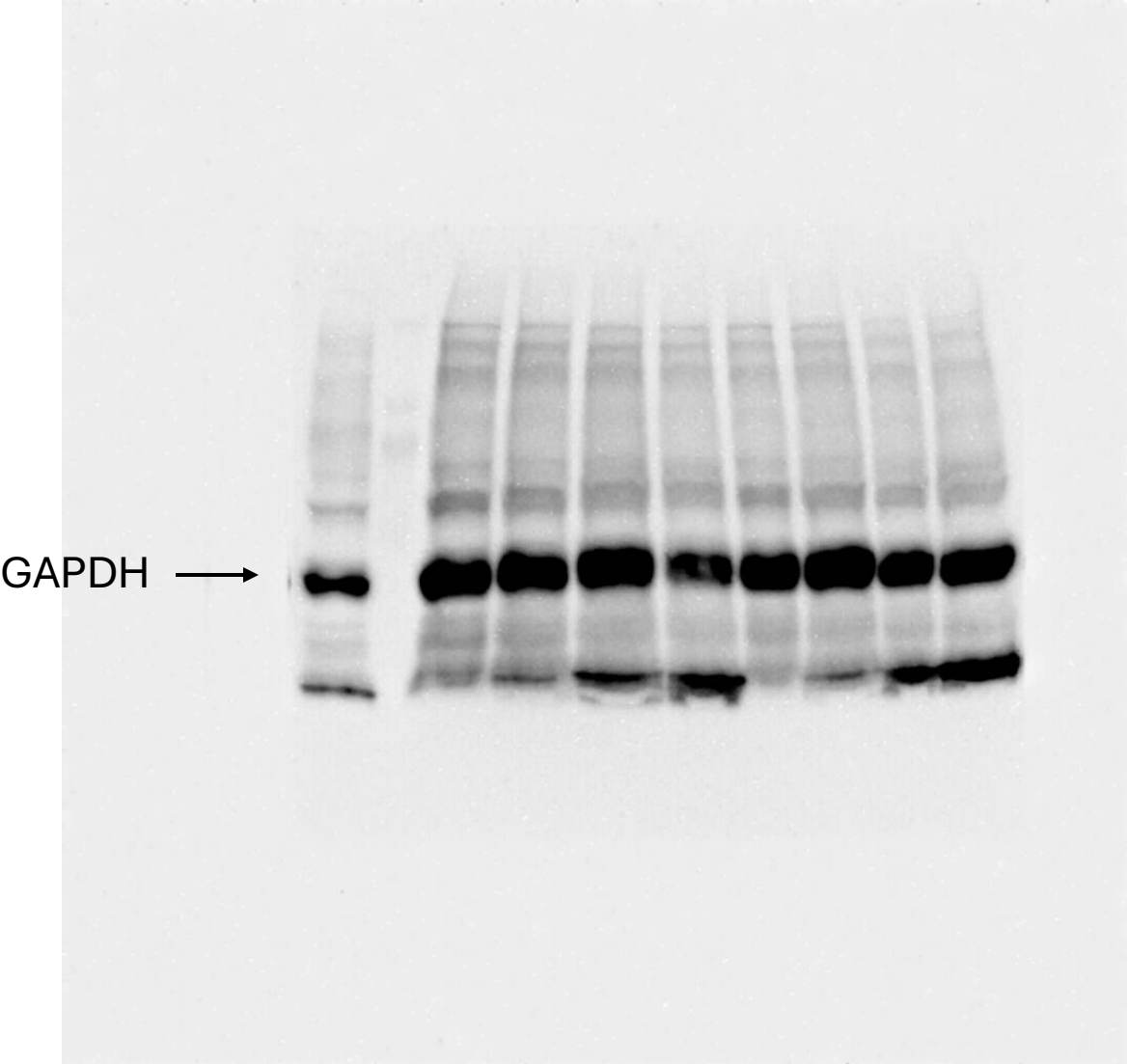

Full unedited gel for Figure 7D

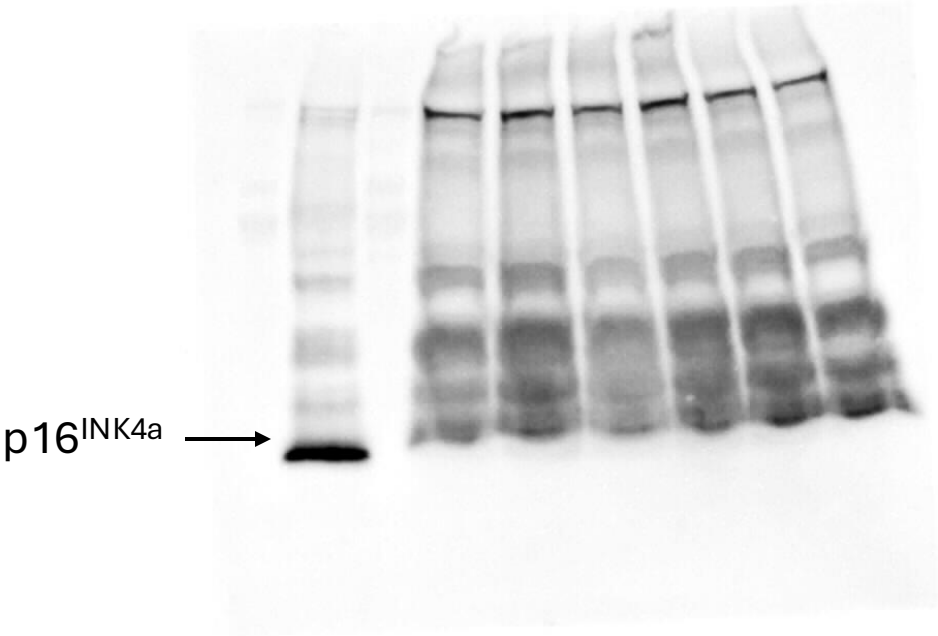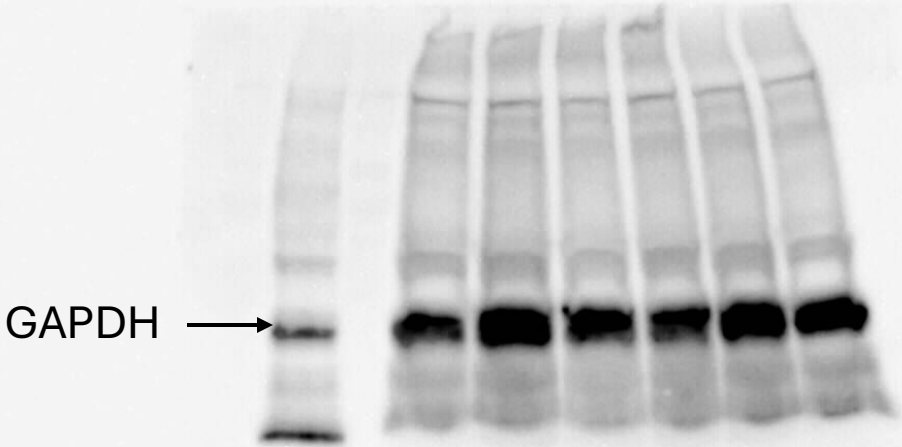

Supplement: Unedited blot and gel images [file jciinsight-10-182103-s206.pdf]
